# Multiple Mechanisms Converging on Transcription Factor EB Activation by the Natural Phenol Pterostilbene

**DOI:** 10.1155/2021/7658501

**Published:** 2021-12-28

**Authors:** Martina La Spina, Michele Azzolini, Andrea Salmaso, Sofia Parrasia, Eva Galletta, Marco Schiavone, Martina Chrisam, Andrea Mattarei, Giulietta Di Benedetto, Andrea Ballabio, Natascia Tiso, Mario Zoratti, Lucia Biasutto

**Affiliations:** ^1^Department of Biomedical Sciences, University of Padova, Padova, Italy; ^2^CNR Neuroscience Institute, Padova, Italy; ^3^Department of Physiology and Pharmacology, Karolinska Institute, Stockholm, Sweden; ^4^Department of Biology, University of Padova, Padova, Italy; ^5^Department of Molecular and Translational Medicine, University of Brescia, Brescia, Italy; ^6^Department of Molecular Medicine, University of Padova, Padova, Italy; ^7^Department of Pharmaceutical and Pharmacological Sciences, University of Padova, Padova, Italy; ^8^Telethon Institute of Genetics and Medicine (TIGEM), Pozzuoli, Italy; ^9^Department of Translational Medical Sciences, Section of Pediatrics, Federico II University, Naples, Italy; ^10^Department of Molecular and Human Genetics, Baylor College of Medicine, Houston, TX, USA; ^11^Jan and Dan Duncan Neurological Research Institute, Texas Children Hospital, Houston, TX, USA

## Abstract

Pterostilbene (Pt) is a potentially beneficial plant phenol. In contrast to many other natural compounds (including the more celebrated resveratrol), Pt concentrations producing significant effects *in vitro* can also be reached with relative ease *in vivo*. Here we focus on some of the mechanisms underlying its activity, those involved in the activation of transcription factor EB (TFEB). A set of processes leading to this outcome starts with the generation of ROS, attributed to the interaction of Pt with complex I of the mitochondrial respiratory chain, and spreads to involve Ca^2+^ mobilization from the ER/mitochondria pool, activation of CREB and AMPK, and inhibition of mTORC1. TFEB migration to the nucleus results in the upregulation of autophagy and lysosomal and mitochondrial biogenesis. Cells exposed to several *μ*M levels of Pt experience a mitochondrial crisis, an indication for using low doses in therapeutic or nutraceutical applications. Pt afforded significant functional improvements in a zebrafish embryo model of ColVI-related myopathy, a pathology which also involves defective autophagy. Furthermore, long-term supplementation with Pt reduced body weight gain and increased transcription levels of *Ppargc1a* and *Tfeb* in a mouse model of diet-induced obesity. These *in vivo* findings strengthen the *in vitro* observations and highlight the therapeutic potential of this natural compound.

## 1. Introduction

Macroautophagy (degradative/lysosomal autophagy; in this paper “autophagy,” for short) is a tightly regulated cell-degradative process leading to the removal of cellular components through lysosomes. It can be activated in response to stress conditions, such as nutrient deprivation, or to degrade and recycle damaged macromolecules and organelles, and it is crucial for the maintenance of cell homeostasis. Autophagy may lead to cell death under specific circumstances (i.e., tumor suppression) [1–3], and it has been shown to contribute to the pathogenesis of different diseases when it is not carried out properly [4–11]. One example are Collagen VI muscular dystrophies [12, 13]: various components of the extracellular matrix (ECM) have been found to regulate autophagy, and this partly explains pathological phenotypes associated with ECM defects [14, 15]. On the other hand, an example of autophagy-mediated protective action is given by redox-active toxicants such as Cr^VI^ [16, 17], which induce mitochondrial dysfunction and organ damage mediated through generation of excessive amounts of reactive oxygen species (ROS). In these cases, autophagy helps cell survival by eliminating malfunctioning mitochondria, a process called “mitophagy,” which also intervenes in other cases, such as neurodegeneration and cardiac myopathies [18–20]. ROS, involved in cell death in this example, are also autophagy inducers [21, 22].

The major controller of lysosomal biogenesis and autophagy may be considered to be transcription factor EB (TFEB), one of four related transcription factors of the MiT-TFE family. TFEB cellular localization and functions are tightly regulated by posttranslational modifications downstream of multiple molecular signaling. One of the best characterized TFEB-regulating mechanisms consists of its recruitment at the lysosomal surface by direct binding to Rag GTPase heterodimeric complexes, followed by inhibitory phosphorylation events by mTORC1 at several serine residues, responsible for TFEB sequestration in the cytosol by 14-3-3 proteins. However, in case of nutrient paucity as well as of severe and prolonged cellular stress (e.g., oxidative stress), both Rag GTPase complexes and mTORC1 are no longer active, while TFEB activating signals such as Ca^2+^-dependent Calcineurin activation prevail, leading to TFEB dephosphorylation and migration to the nucleus, where it sets in motion the transcriptional program for Coordinated Lysosomal Expression And Regulation (the CLEAR network) [23–28] (Suppl. Figure 1). Analogous functions are carried out by TFE3, another member of the family [29].

The involvement of autophagy in multiple relevant biological processes has made it a prime pharmacological target [30–34], but the need for specific autophagy-targeting drugs remains.

An acute, pharmacologically induced, up- or down-regulation of autophagic flux may be desirable in some pathological contexts—e.g., sepsis [35] or cancer [33, 36]. In many other conditions (e.g., aging, neurodegeneration, muscular dystrophy, and metabolic disorders), however, a sustained moderate upregulation would be beneficial, for example, by helping to prevent the formation of protein aggregates (a hallmark of neurodegeneration) [30, 37]. Such a “soft” effect is typical of bioactive natural products present in a balanced human diet. *In vitro* evidence of autophagy induction has been obtained with several dietary plant phenols; one of the best-documented examples is that of resveratrol [38–45]. While celebrated for their multiple beneficial activities, most plant phenols are however affected by issues of bioavailability and metabolism when used *in vivo* [46–48], being rapidly and extensively modified by Phase II enzymes and by the intestinal flora. As a consequence, concentrations used in *in vitro* studies often largely exceed the levels reached by these compounds *in vivo*.

Pterostilbene (Pt, 3,5-di-O-methylresveratrol) [49–52] is much more bioavailable than resveratrol [53–56] due to its higher lipophilicity and to the presence in the molecule of only one hydroxyl group. Levels of several nmoles/gr can be reached and maintained for hours in some organs (e.g., liver and brain) upon oral administration of a moderate pharmacological dose of Pt (88 *μ*moles/Kg body weight) [56]. This may account at least in part for its asserted higher efficacy [57–59]. Induction of autophagy is one of the effects of Pt [60–63].

Given the strong biomedical implications, we set out to investigate the mechanisms underlying this activity, and to test its potential usefulness *in vivo*. Using a HeLa cell line overexpressing a TFEB-GFP chimera, we first verified that Pt could induce TFEB translocation, then sought to characterize the signaling cascades prompting it. We used a range of concentrations (1, 10, and 25 *μ*M) that were shown to be achieved in rodents after ingestion of Pt [56, 64].

To consolidate the notion that Pt may be beneficial in pathologies affected by a deficit of autophagy, we used two models. In the first, expression of Collagen VI was downregulated by injecting morpholino oligomers in zebrafish embryos. Collagen VI myopathies are characterized by defective autophagy, whose restoration can rescue the dystrophic phenotype [65]. The second was a murine model of diet-induced obesity, in which we had already observed a “slimming” effect of long-term Pt administration [64]. Also in this context, autophagy is thought to counteract obesity and obesity-associated diabetes [66]. In both cases the results were coherent with a Pt-induced, autophagy-mediated, health-promoting effect.

## 2. Materials and Methods

### 2.1. Cell Cultures

Migration experiments were carried out in HeLa cells overexpressing a TFEB-GFP construct, generated as described by Settembre et al. [67]. Cells were cultured in high-glucose Dulbecco's Modified Eagle Medium (DMEM, Sigma-Aldrich) supplemented with 10% Fetal Bovine Serum (FBS, Euroclone), 1% penicillin/streptomycin (10000 U/mL and 10 mg/mL, respectively) and Geneticin 418 (G418, 100 *μ*g/mL, Sigma-Aldrich).

Western blot, RT-qPCR analysis, and ROS, calcium, mitochondrial depolarization, and mitochondrial mass analysis were performed with wild-type (WT) HeLa cells, maintained in high-glucose DMEM supplemented with 10% FBS and 1% penicillin/streptomycin.

For microscopy, 200,000 cells were seeded over a glass coverslip (24 mm diameter, BDH) one day before the experiment and incubated at 37°C to allow adhesion. Likewise, cells were plated on plastic multiwells, at the same confluency, for any other measurement.

### 2.2. Drugs and Treatments

Treatments with Pt (Waseta Int. Trading Co., Shangai, P.R. China) were carried out at 1, 10, and 25 *μ*M final concentrations. Additional drugs used were A769662 (Abcam), Forskolin (Sigma-Aldrich) and 3-isobutyl-1-methylxanthine (IBMX, Abcam), N-Acetyl-L-Cysteine (NAC, Sigma-Aldrich), carbonyl cyanide 4-(trifluoromethoxy) phenylhydrazone (FCCP, Sigma-Aldrich), 2-[2-(3,4-Dihydro-2,2,4-trimethyl-1(2H)-quinolinyl)-2-oxoethyl]-1H-isoindole-1,3(2H)-dione (MLS-A1, Tocris Bioscience), cAMP (Sigma-Aldrich), ATP (Sigma-Aldrich), N-methyl-4-isoleucine cyclosporin (NIM811), 1,2-Bis(2-aminophenoxy)ethane-N,N,N′,N′-tetraacetic acid tetrakis (acetoxymethyl ester) (BAPTA-AM, Sigma-Aldrich), cyclopiazonic acid (CPA, Sigma-Aldrich), and ethylene glycol-bis(2-aminoethylether)-N,N,N′,N′-tetraacetic acid (EGTA, Sigma-Aldrich). Stock solutions of the compounds were prepared at 1000× concentration to set the final vehicle percentage at 0.1% in all cases (including controls without drugs). Treatments were performed in media with no FBS and phenol red to avoid interference with drugs.

### 2.3. Migration Experiments

HeLa cells overexpressing TFEB-GFP seeded on glass coverslips were mounted in an appropriate holder and gently rinsed with PBS. 1 mL of Leibovitz's L-15 medium (Life Technologies) was finally added. This buffered medium is designed for supporting cell growth in low-CO_2_ environments or without CO_2_ equilibration, and therefore it was preferred over DMEM. A SP2 confocal (Leica) or a DM6000 inverted (Leica) microscope were used to follow migration kinetics. TFEB-GFP migration was monitored in real-time for up to 3 hours after the addition of the desired compound. Positive control was represented by starvation (i.e., cells were incubated in HBSS instead of culture medium). Excitation was at 460 nm and fluorescence was collected in at *λ* > 510 nm. Images were taken every 15 minutes, using 40× oil immersion objectives. The instruments were equipped with a thermostat set at 37°C. ImageJ software was used to measure TFEB migration to the nucleus. A few circular fields (regions of interest, ROIs) were drawn within the area of the nuclear and cytosolic cross sections of a given cell and their mean fluorescence pixel intensity reading was used to calculate nucleus to cytosol ratio. All ratios were normalized to the value at time 0.

### 2.4. Superoxide Generation and Mitochondrial Membrane Potential Measurements

WT HeLa cells seeded on glass coverslips were gently rinsed once with PBS and then loaded with 1 *μ*M MitoSOX™ Red or 20 nM TMRM in DMEM (Thermo Fisher Scientific), in the presence of 0.8 *μ*M Cyclosporine H (CsH, Sigma-Aldrich), at 37°C for 30 minutes in the darkness. Experiments were then performed in Leibovitz's L-15 medium. Images were taken every 2 minutes with a 40× oil immersion objective, using a Leica DM6000 fluorescence microscope. Excitation was at 488 nm, and fluorescence was collected at *λ* > 570 nm. Kinetics were followed for up to 90 minutes. ImageJ software was used to draw ROIs along the cells' perimeter and to calculate their relative fluorescence intensity over time.

### 2.5. Calcium Measurements

WT HeLa cells seeded on glass coverslips were gently rinsed with PBS and loaded with 0.5 *μ*M Fluo-4 AM (Thermo Fisher Scientific) in DMEM, in the presence of 0.8 *μ*M CsH, at 37°C for 30 minutes in the darkness. Fluo-4 fluorescence kinetics were followed for up to 30 minutes. Images were captured every 5 seconds using a Leica DM6000 microscope. Excitation was at 460 nm, and fluorescence was collected at *λ* > 510 nm. Quantification was performed as described above for MitoSOX™ and TMRM signals. Calcium imaging was also performed using Fura-2 AM (Thermo Fisher Scientific): cells were gently rinsed with PBS and loaded with 0.5 *μ*M Fura-2 AM in an extracellular-like saline solution (in mM: 135 NaCl, 5 KCl, 0.4 KH_2_PO_4_, 1 MgCl_2_, 20 HEPES, 10 glucose, 1 CaCl_2_, pH 7.4), in the presence of 0.02% Pluronic F-127 and 200 *μ*M sulfinpyrazone [68], for 20 minutes at 37°C. Cells were further incubated in saline (without additions) for 20 minutes at 37°C, Pt or other drug/medium was added, and then fluorescence kinetics were followed in real time for up to 30 minutes. Images were captured every 5 seconds using an inverted microscope (Zeiss Axiovert 100). Excitation was alternatively at 340 and 380 nm, and fluorescence was collected at 500–530 nm. ROIs, corresponding to single cells, were selected for Ca^2+^ imaging. Mean fluorescence intensity measured at the two excitation wavelengths for each ROI was subtracted of the background. The ratio of the emitted fluorescence intensities (R_340/380_ = F_340_/F_380_) at each time point was normalized to the value measured at the beginning of the experiment.

### 2.6. Western Blots

Cells were lysed in Laemmli buffer (2% SDS, 62.5 mM Tris-HCl pH 6.8, 10% Glycerol, 50 mM DTT) supplemented with fresh protease and phosphatase inhibitors cocktails 2 and 3 (Sigma-Aldrich). The lysate was kept in ice for 30 minutes to achieve the total brakeage of cell membranes, and centrifuged (12,000 g, 20 min, 4°C). The resulting supernatant was assessed for protein content using the BCA assay (Thermo Fisher Scientific). Prior to electrophoresis, samples were supplemented with sample buffer (2% SDS, 62.5 mM Tris-HCl pH 6.8, 50 mM DTT, 10% glycerol, 0.01% bromophenol blue) and heated before loading. Pre-cast Bis-Tris gels (NUPAGE, Thermo Fisher Scientific) at different percentages of acrylamide were used depending on the molecular weight of the proteins of interest. After electrophoretic separation, proteins were transferred to PVDF membranes (Immobilion-FL). The membranes were saturated with 5% BSA (Sigma-Aldrich) at room temperature for 1 hour and incubated with primary antibodies overnight at 4°C. The following day, the membranes were washed with TBS Tween 0.1% (TBST) and incubated with IRDye 680RD or 700DX secondary antibodies (LICOR) or horseradish peroxidase-conjugated secondary antibodies (Cell Signaling). Finally, membranes were washed again with TBST, and protein signal was detected with the Odissey Imaging System (LICOR) or with chemiluminescence detection (Pierce) using digital imaging by a UVITEC Eppendorf apparatus.

### 2.7. RT-qPCR Analyses

Total RNA was extracted from cells using TRIzol (Thermo Fisher Scientific). Reverse transcription was performed by using the SuperScriptVILO cDNA synthesis Kit (Thermo Fisher Scientific). Reaction mix tubes were shaken and incubated first at 25°C for 10 minutes and then at 42°C for 60 minutes. Finally, the reaction was arrested by heating at 85°C for 5 minutes. All primers were purchased from Sigma-Aldrich and their sequences are listed in Supplementary Table 1. IQ Syber Green Supermix (Biorad) was used as detection system. ∆∆Ct value was calculated between the controls and the treated samples. Lastly, the fold change was calculated using 2^(-∆∆Ct)^ [69]. *ACTB* or *Gapdh* were used as reference genes.

### 2.8. Respiratory Chain Complex I and Complex III Activities

NADH-CoQ oxidoreductase (complex I) and CoQ cytochrome c oxidoreductase (complex III) activities were assayed using permeabilized Rat Liver Mitochondria (RLM), as described by Sassi et al., 2014 [70].

### 2.9. Flow Cytometric Analysis of Mitochondrial Content

WT HeLa cells were detached, centrifuged (200 g, 10 minutes), resuspended in DMEM and stained with 2 *μ*M Acridine Orange 10-nonyl bromide (NAO, Sigma-Aldrich) at 37°C for 30 minutes in darkness (1,5 × 10^6^ cells/mL). After loading, cells were resuspended in HBSS (plus 1 *μ*M CsA, 3 × 10^5^ cells/mL) and divided into identical aliquots. A Beckton Dickinson II flow cytometer was used, and 10,000 events were counted for each measurement. Excitation was at 488 nm and fluorescence was collected in the 542–585 nm interval. Data were analyzed using the BD VISTA software. Averages ± s.d. of the medians of fluorescence distribution histograms were plotted, normalized to the value measured for the control.

### 2.10. Zebrafish Model of ColVI Deficiency

Adult WT fish were maintained in the facility of the University of Padova in tanks containing aerated, 28.5°C thermostatted saline water according to standard protocols. Fish were kept in a regimen of 14 hours of light alternated by 10 hours in the dark. Eggs were collected and kept in fish water (0.5 mM NaH_2_PO_4_, 0.5 mM Na_2_HPO_4_, 0.2 mg/L methylene blue, 3 mg/L “Instant ocean sea salt”) at 28.5°C. All zebrafish procedures were performed under UniPD Ethical Committee (OPBA) authorization 407/2015-PR.

#### 2.10.1. Injection with Antisense Morpholinos

To target ColVI protein we took advantage of a morpholino oligomer designed to skip exon 9 of zebrafish *col6a1* gene, previously designed by Telfer et al., 2010 [71], leading to translation of a truncated, dominant negative, version of the ColVI alpha1 chain. Embryos from WT incrosses were injected with the morpholino (about 4 ng per embryo) at the 1-4 cell stage using a WPI pneumatic PicoPump PV820 injector.

#### 2.10.2. Drug Treatments

Zebrafish embryos were dechorionated at 20 hpf (hours postfertilization) and exposed to Pt treatment at 24 hpf in Petri dishes. Preliminary experiments were performed on WT embryos to choose the most suitable dosage for the treatments: Pt was diluted in fish water at different concentrations (ranging from 0.05 to 10 *μ*M), and touch-evoked escape response (see below) was evaluated at 48 hpf (data not shown). Subsequent experiments were performed incubating embryos with 1 *μ*M Pt, that turned to be the optimal concentration.

#### 2.10.3. Motor Activity Test

Spontaneous coiling rate was recorded as the number of events observed in 15 seconds for each embryo at 24 hpf using a light microscope. For the touch escape response assay, we observed the ability of embryos at 48 hpf to escape after a brief touch with a small tip. Individuals were classified into four groups according to their ability to escape: 3, normal (corresponding to normal swimming motility); 2, motor impairments (embryos with minor motility abnormalities); 1, only coiling events (embryos circling without the ability to escape) and 0, paralyzed (embryos with no motility).

#### 2.10.4. Birefringence Assay

Muscle birefringence was analyzed by exploiting muscle fibers anisotropy. Briefly, fish were anesthetized with tricaine (160 *μ*g/mL) and placed on a glass slide. Two polarizing filters were used. The first filter polarizes light of the stereomicroscope (Leica M165FC). This polarized ray is refracted by anisotropic muscles. The second filter (analyzer) is finally twisted to detect the angle of refracted light until the muscular fibers become maximally brilliant.

We calculated the density integrated area of birefringence using ImageJ software, as described by Berger et al., 2012 [72]. Birefringence values ≥2 × 10^6^ (typical of WT individuals) were rated as normal, values between 2 × 10^6^ and 1 × 10^6^ were considered as an indication of mild disease and values ≤1 × 10^6^ were rated as an indication of severe myopathy.

#### 2.10.5. Protein Extraction

48 hpf zebrafish embryos were deyolked in cold PBS supplemented with 1 mM PMSF on ice. Tissues were then frozen in liquid nitrogen. Samples were homogenized in Tissue Extraction Reagent (Thermo Fisher Scientific), supplemented with fresh protease and phosphatase inhibitors cocktails 2 and 3 (Sigma-Aldrich) using a motor pestle and centrifuged (12000 g, 20 min, 4°C). Finally, supernatant was used for Western blot (see the protocol above).

### 2.11. Mouse Model of Diet-Induced Obesity

C57BL/6 mice were housed in the facility of the Department of Pharmacological Sciences (Padova); food and water were provided ad libitum. Procedures were all approved by the University of Padova Ethical Committee for Animal Welfare (OPBA) and by the Italian Ministry of Health (Permit Number 211/2015-PR), and conducted with the supervision of the Central Veterinary Service of the University of Padova, in compliance with Italian Law DL 26/2014, embodying UE Directive 2010/63/EU.

#### 2.11.1. Animal Treatments

The cohort of mice was the same considered in [64]. Briefly, after weaning obese mice (HFD group) were fed a high fat diet (60% calories from fat), while Pt-treated (HFD + Pt) mice were fed the high fat diet supplemented with Pt (90 mg/Kg body weight/day). Mice were maintained under these dietetic regimens for 30 weeks; at the end of this period, they were sacrificed after being fasted for 4 hours. Inguinal adipose tissue was collected, immediately frozen in liquid nitrogen and then stored at -80°C until analysis. RNA extraction and RT-qPCR were performed as described above and in [64]. Primer sequences are reported in Supplementary Table 1.

### 2.12. Statistics

Two-way ANOVA analysis for repeated measures as a function of time and treatment was performed to assess differences in TFEB migration, superoxide generation and mitochondrial depolarization experiments. One-way ANOVA analysis was used for TFEB migration at 3 hours, complex I activity and NAO fluorescence. Dunnett's, Tukey's or Sidak's corrections were applied to compare drug efficacies versus control only, with respect to each other, or with respect to only some specific conditions, respectively. When considering Western Blots, significance in comparisons was evaluated by applying Kruskal-Wallis' non-parametric test, with Dunn's correction. RT-qPCR data were analyzed performing Student's *t*-test or one-way ANOVA (with Dunnett's correction). The efficacy of Pt treatment with respect to untreated ColVI-exon9 zebrafish “morphants” (morpholino-injected) in recovering motor abilities and myofibers structure of ColVI-exon9 zebrafish morphants was evaluated by applying Student's *t-*test. GraphPad Prism software was used to perform all the tests mentioned above. Significance in comparisons is indicated in the figures as follows: ∗*p* < 0.05; ∗∗*p* < 0.01; ∗∗∗*p* < 0.001; ∗∗∗∗*p* < 0.0001.

## 3. Results

### 3.1. TFEB Migration

We confirmed that Pt induces autophagy *in vitro* at low concentrations: lipidated LC3 and LC3-positive puncta increased in WT HeLa cells treated with 1-25 *μ*M Pt (Suppl. Figure 2).

**Figure 1:**
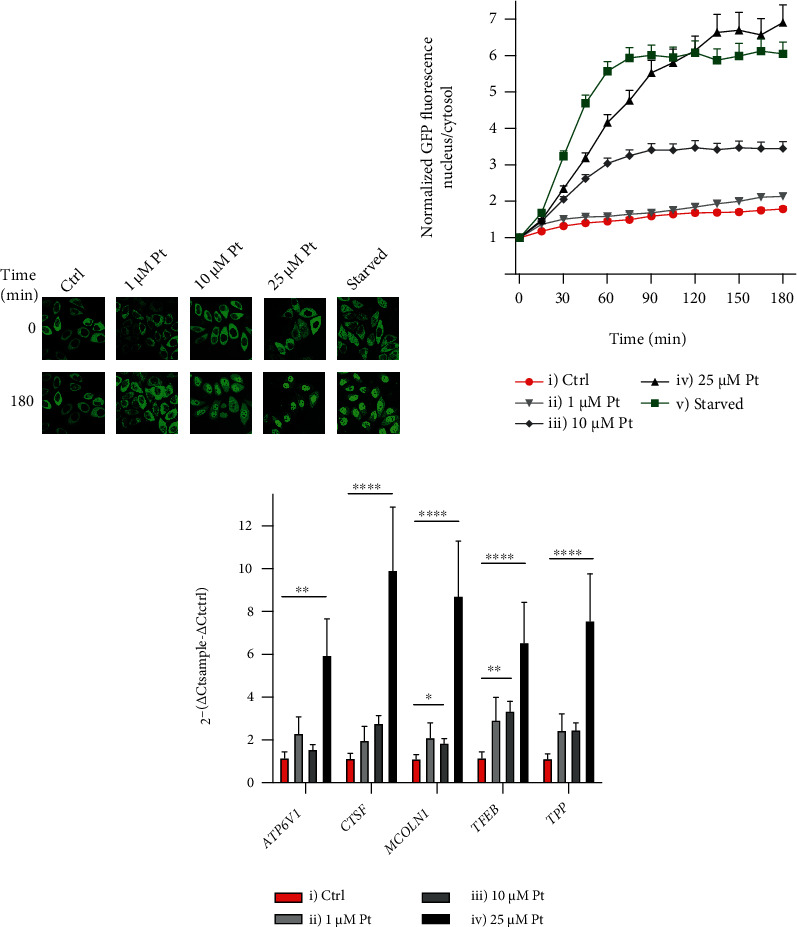
Pt induces translocation and activation of TFEB in HeLa cells. TFEB migration kinetics: (a) representative images of HeLa cells overexpressing TFEB-GFP, treated as indicated, and (b) plot of the nucleus/cytosol green fluorescence ratios after the indicated additions or medium exchange at time zero. Mean values + SEM; *N* ≥ 55 cells for each time point and condition, observed in at least 3 separate experiments. Comparison with Ctrl: *p* < 0.0001 from 15 min for curves (iii), (iv), and (v); *p* < 0.05 from 135 min for curve (ii). (c) Pt enhances the transcription of TFEB and its target genes. Mean values + SEM; *N* ≥ 5. Horizontal bar extremities correspond to the columns to be compared.

Using HeLa cells overexpressing a TFEB-GFP fusion protein [67], we demonstrated that Pt causes TFEB-GFP migration to the nucleus (Figures 1(a) and 1(b)). Over a period of 3 hours, 25 *μ*M Pt was about as effective as starvation (lack of amino acids). 1 *μ*M Pt induced only a weak – but significant (after 135 min) – migration, while 10 *μ*M Pt elicited an intermediate behavior. Coherently, we observed a concentration-dependent increase in the transcription levels of a set of TFEB target genes, including *TFEB* itself, *MCOLN1* (an endosomal/lysosomal channel), *ATP6V1* (a subunit of a lysosomal proton pump), *TPP1* and *CTSF* (two proteolytic enzymes) [73] (Figure 1(c)).

### 3.2. Superoxide Generation

**Figure 2:**
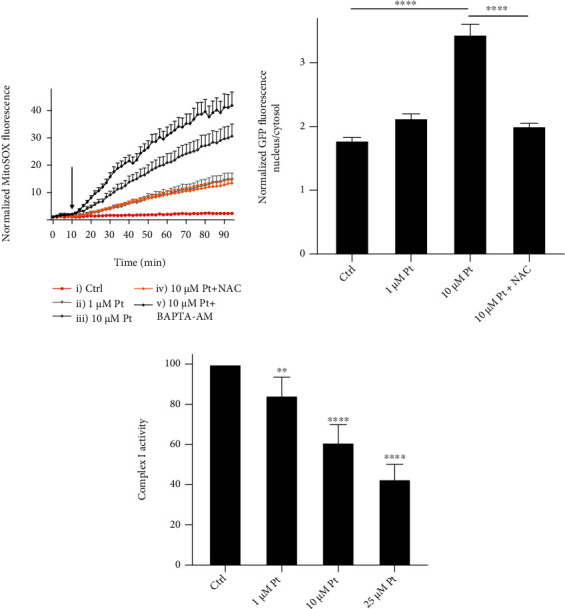
Pt elicits mitochondrial superoxide generation in HeLa cells. (a) MitoSOX™ fluorescence response elicited by Pt. The cells in curves iv) and v) were pretreated, respectively, with 1 mM NAC (60 min) or 5 *μ*M BAPTA-AM (20 min), and continued to be incubated with these agents also during treatment with Pt. The arrow indicates the addition of Pt. Mean values + SEM. (b) NAC antagonizes Pt-induced TFEB migration to the nucleus. TFEB migration in HeLa cells exposed to the indicated conditions for 3 hours. Mean values + SEM. *N* > 53 cells for each tested condition, from at least 4 separate experiments. (c) Pt inhibits Complex I activity in permeabilized RLM. Mean values + SEM; *N* = 6.

Several studies have pointed to ROS, and especially to mitochondrial ROS, as key mediators of autophagy [21, 22, 74–77]. We observed a Pt-elicited increase of the signal of the mitochondria-targeted superoxide probe MitoSOX™ (Figure 2(a), Suppl. Figure 3), and a Pt-induced inhibition of the electron transfer activity of complex I (Figure 2(c)). No effect was observed on the activity of complex III (data not shown). The combination of 25 *μ*M Pt and MitoSOX™, however, proved cytotoxic, preventing the collection of reliable data. It has indeed been reported that MitoSOX™ – at concentrations much higher than the ones we used – can be cytotoxic [78] and the presence of 25 *μ*M Pt enhances this toxicity.

Pretreatment of the cells with the antioxidant NAC reduced the MitoSOX™ response (Figure 2(a), trace iv), and TFEB migration to the nucleus (Figure 2(b)) to levels similar to those induced by 1 *μ*M Pt (Figure 2(a), trace ii, and Figure 2(b)).

### 3.3. mTORC1 Inhibition

**Figure 3:**
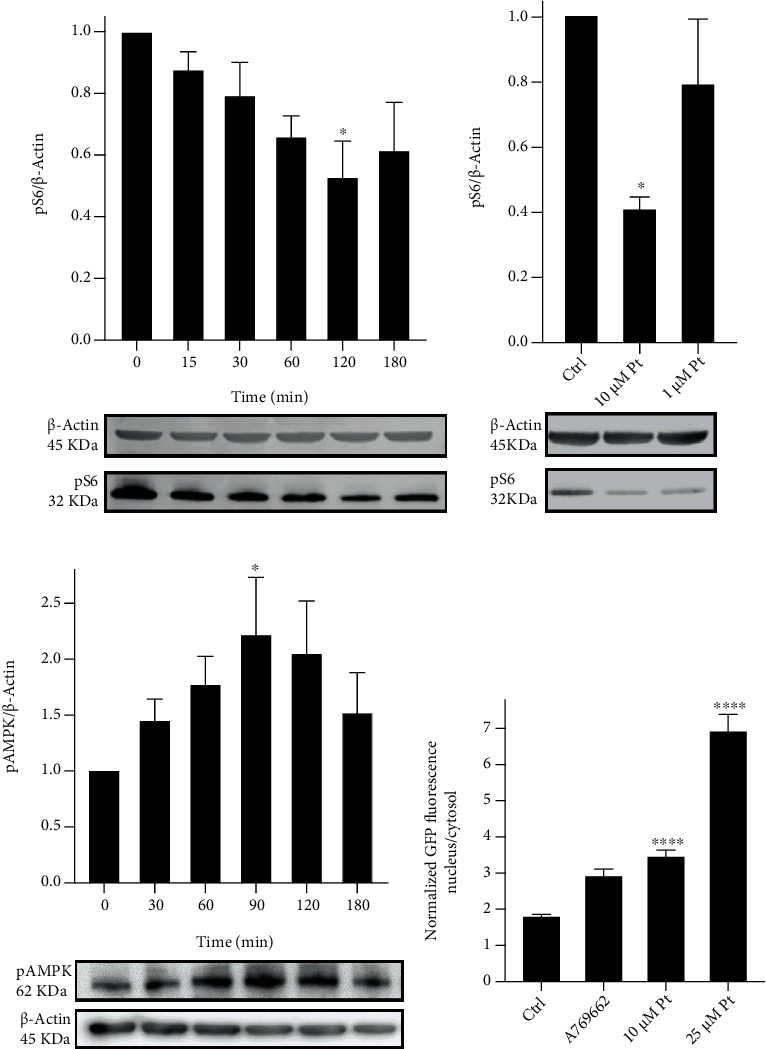
(a, b) Pt determines mTORC1 inhibition in HeLa cells. Western blot analysis of phospho-S6 (S240/244). (a) Time-dependent reduction of S6 phosphorylation by 25 *μ*M Pt. (b) Effect on S6 phosphorylation of a 2 h treatment with 10 or 1 *μ*M Pt. Representative Western blot images are shown below the histograms. Mean values + SEM; *N* ≥ 4. (c, d) AMPK is activated by Pt in HeLa cells. (c) Western blot analysis of phospho-AMPK (T172). Time-dependent increase in AMPK phosphorylation by 25 *μ*M Pt. Representative Western blot images are shown below the histograms. Mean values + SEM; *N* ≥ 4. (d) A partial TFEB-GFP migration is elicited by pharmacological AMPK activation. TFEB migration in HeLa cells exposed to 25 and 10 *μ*M Pt (same data as in Figure 1(b)) or to 25 *μ*M A769662 for 3 hours. Mean values + SEM. *N* ≥ 20 cells for each condition, observed in at least 3 separate experiments.

Current paradigms in the field state that starvation-induced TFEB migration is mainly determined by its dephosphorylation, downstream of mTORC1 inhibition and Ca^2+^-elicited calcineurin activity. We tested whether these processes were involved also in Pt-induced TFEB migration. Indeed, treatment of HeLa cells with Pt determined mTORC1 inhibition, as deduced from the decreased phosphorylation of the ribosomal protein S6, one of its major targets (Figures 3(a) and 3(b)). After two hours of incubation, the effect was significant when applying 25 or 10 *μ*M Pt. With 1 *μ*M significance was not achieved, but the same trend was observed.

### 3.4. AMPK Activation

mTORC1 can be inhibited via phosphorylation by AMPK (see Suppl. Figure 1), and indeed migration of TFEB to the nucleus could be induced by A769662, an AMPK activator [79] (Figure 3(d)). Translocation was less pronounced than when induced by starvation or by 25 or even 10 *μ*M Pt (Figure 3(d)). This indicates that the AMPK-comprising branch of the signaling cascades can only partially account for the extent/kinetics of migration observed with 10 or 25 *μ*M Pt. Activating phosphorylation of AMPK, indeed, took place in cells exposed to 25 *μ*M Pt, as shown by Western Blots for phospho-AMPK (Figure 3(c)). However, we were unable to detect significant changes in the levels of this phosphoprotein at the lower Pt concentrations (not shown).

### 3.5. Effects on CREB Signaling

In C2C12 myotubes resveratrol activates AMPK via an increase of cAMP due in turn to inhibition of part of the PDEs [80]. In agreement with the possibility that Pt might also determine a cAMP increase, we recently observed that Pt administration increased the phosphorylation of cAMP Responsive Element Binding protein (CREB) in the hippocampus of aged rats and in HEK293 cells [81]. We confirmed here that also in WT HeLa cells administration of Pt determined an increase of the phosphorylation of CREB, but concluded that this was not linked to an increase of cAMP levels (for details see Suppl. Figure 4 and associated text). Even when an increase in cAMP levels was forced using Forskolin and IBMX, TFEB-GFP did not migrate to any significant extent (Suppl. Figure 4F). These results are in agreement with the conclusion by Park et al. [39] that stilbenoids can elicit autophagy independently of the cAMP pathway. To clarify this point, and because Ca^2+^ is responsible for the activation of calcineurin (Suppl. Figure 1 and references provided there), we investigated if Pt could affect Ca^2+^ signaling.

### 3.6. Ca^2+^ Mobilization

**Figure 4:**
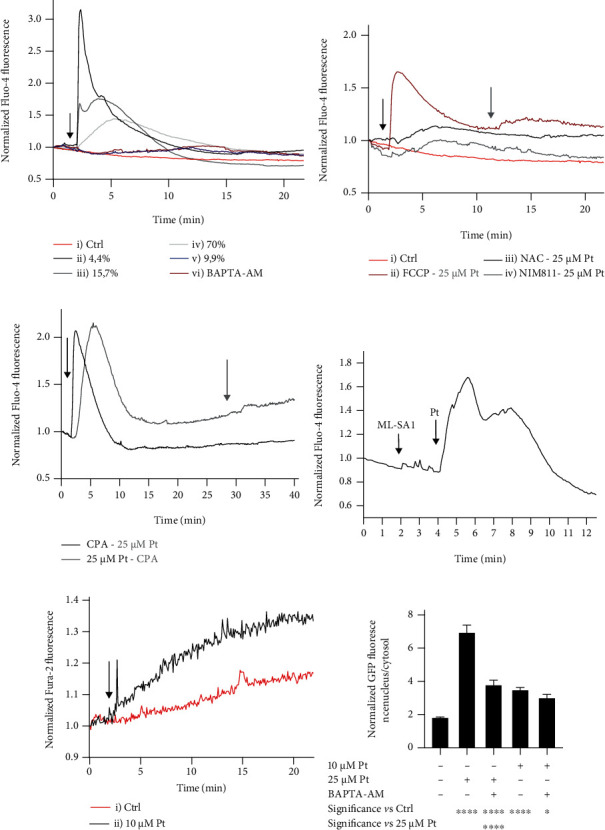
Pt elicits Ca^2+^ signaling in HeLa cells; *N* > 36 for each time point and condition, observed in at least 3 separate experiments. (a) Cytosolic Ca^2+^ signals induced by 25 *μ*M Pt (Fluo-4 fluorescence). The arrow indicates the addition of 0.1% DMSO in curve i) and of 25 *μ*M Pt in curves ii), iii), iv), v), and vi). The cells in curve vi) were pretreated for 20 min and continued to be incubated after Pt addition with 5 *μ*M BAPTA-AM. (b) Cytosolic Ca^2+^ signals elicited by 25 *μ*M Pt after pretreatment of the cells and in the continuing presence of modulators. 25 *μ*M Pt was added when indicated by the black arrow for curves iii) and iv), when indicated by the grey arrow for curve ii). Modulators were as follows: ii) 2 *μ*M FCCP, added when indicated by the black arrow (no preincubation); iii) 10 mM NAC (preincubation, 30 min); iv) 0.8 *μ*M NIM811 (preincubation, 20 min); i) control: addition of DMSO (0.1% final concentration) when indicated by the black arrow. Please note the difference of the Y scale between panels (a) and (b). (c) Ca^2+^ mobilized by Pt largely originates from ER. Curve i): the SERCA inhibitor CPA (20 *μ*M) was added when indicated by the black arrow; Pt (25 *μ*M) when indicated by the grey arrow. Curve ii): the sequence of the additions was the opposite (Pt first). In both cases, excess EGTA was added to the medium 30 sec before the first addition. (d) The cellular Ca^2+^ mobilized by Pt in HeLa cells comes mostly from subcellular compartments other than the lysosomes (see text for details). Normalized Fluo-4 fluorescence. (e) Cytosolic Ca^2+^ signals elicited by 10 *μ*M Pt measured with the ratiometric probe Fura-2. The arrow indicates the addition of: i) 0.1% DMSO; ii) 10 *μ*M Pt. The difference between the two curves is significant (*p* < 0.05) from *t* = 5 min onwards. (a–e) Error bars are omitted for clarity. (f) TFEB migration in HeLa cells exposed to the indicated conditions for 3 hours. Mean values + SEM.

Stimulation of WT HeLa cells with 25 *μ*M Pt was indeed accompanied by Ca^2+^ mobilization: a transient increase in its cytosolic levels began in most cells within seconds of exposure to 25 *μ*M Pt (Figure 4(a)).

When individually considered, the cells reacted to Pt in one of 4 distinct ways (Figure 4(a)). Out of 181 cells analyzed in 6 separate experiments, 9.9% exhibited no or only a low and slowly developing change of the Fluo-4 signal (curve v). 4.4% responded with a prompt increase of the fluorescence, which then declined to approximately basal levels over about 10 minutes (curve ii). 70% exhibited a somewhat delayed and more slowly declining transient (curve iv). The remaining 15.7% showed a combination of behaviors (ii) and (iv) (curve iii). This diversity suggests the existence of cellular populations with different properties even in the same culture well. Preincubation with 5 *μ*M BAPTA-AM resulted, as expected, in the near-disappearance of the fluorescent response (curve vi).

The cytosolic Ca^2+^ increase indeed seems to be elicited by ROS, since it is antagonized by the antioxidant NAC (Figure 4(b), curve iii), which quenches the MitoSOX™ response (Figure 2(a), curve iv). On the contrary, ROS production is not primarily downstream of the cytosolic Ca^2+^ signal, since suppression of the latter with BAPTA-AM (Figure 4(a), curve vi) did not decrease the MitoSOX™ response (Figure 2(a), curve v; curve v is not significantly different from curve iii).

The magnitude of the signal suggested that Ca^2+^ was being released from the ER, as supported by the results presented in Figure 4(c). Indeed, when the ER is previously emptied by treatment with the SERCA inhibitor CPA, 25 *μ*M Pt failed to induce a significant Fluo-4 fluorescence signal (Figure 4(c), curve i). Conversely, the addition of CPA after 25 *μ*M Pt elicited only a minor Ca^2+^ signal, confirming that the reticular depot had been depleted (Figure 4(c), curve ii). Extensive evidence indicates that the ER and mitochondria are functionally coupled and behave as distinct components of one cellular Ca^2+^-handling system [82–85]. The observation of two clearly different kinetic patterns suggests that the events elicited by Pt may be more complex than Ca^2+^ release via the IP_3_R. Indeed, if the cells are pretreated with NIM811, a mitochondrial permeability transition inhibitor, the Ca^2+^ signal is significantly dampened (Figure 4(b), curve iv). The cytosolic Ca^2+^ transient observed upon addition of the mitochondrial uncoupler FCCP is similar in amplitude to that elicited by 25 *μ*M Pt in most of the cells. Medina et al. [86] have identified lysosomal Ca^2+^ as the relevant pool for starvation-induced TFEB migration. We can however discount an exclusively lysosomal origin of Ca^2+^ in our case. As shown in Figure 4(d), the release of lysosomal Ca^2+^ by the MCOLN-1 activator ML-SA1 did not significantly affect the transient signal elicited by the subsequent addition of 25 *μ*M Pt. Obviously, this does not imply that Pt does not mobilize also lysosomal Ca^2+^. In any case, we see no contradiction between the present results and those reported in [86] since a strong and prolonged increase of cytosolic Ca^2+^ is likely to extend also to any microdomains that in other circumstances (starvation) may sense only the Ca^2+^ released by the lysosomes. In our hands, addition of only ML-SA1 to TFEB-GFP-expressing cells under control conditions promptly induced migration of the transcription factor to the nucleus (Supplementary Figure 5), confirming the results of Medina et al. [86]. Preincubation with the permeant Ca^2+^ chelator BAPTA-AM completely abolished the effect (Supplementary Figure 5).

At lower Pt concentrations (1 or 10 *μ*M Pt) the intensiometric probe Fluo-4 did not provide reliable indications. We therefore used the ratiometric probe Fura-2-AM. Fura-2 allowed us to conclude that indeed 10 *μ*M Pt also elicited a cytosolic Ca^2+^ signal (Figure 4(e)). This consisted of a less marked but prolonged increase.

Ca^2+^ indeed may play a major role for TFEB-GFP translocation in our experimental system (Figure 4(f)). Interestingly, in the presence of BAPTA a similar distribution of TFEB-GFP between the nucleus and the cytosol was eventually reached with either 25 or 10 *μ*M Pt (Figure 4(f)). The convergence to similar nucleus/cytosol TFEB ratios suggests the intervention of migration-inducing Ca^2+^/CaM-independent signaling elicited by Pt at concentrations ≤10 *μ*M.

### 3.7. Impact on Mitochondria

**Figure 5:**
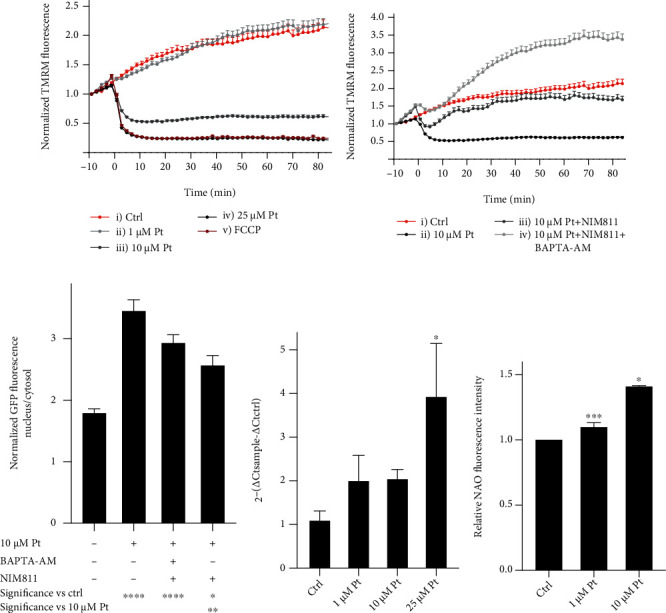
Impact of Pt on mitochondria. (a, b) Pt induces mitochondrial depolarization. Normalized fluorescence intensity of TMRM-loaded HeLa cells, treated as indicated. (c) TFEB migration (expressed as nuclear/cytosolic fluorescence ratio) in HeLa cells exposed to the indicated conditions for 3 hours. Mean values + SEM. *N* ≥ 20 cells for each time point and condition, observed in at least 3 separate experiments. (d, e) Pt increases mitochondrial biogenesis in WT HeLa cells. (d) RT-qPCR analysis of *PPARGC1a*. *N* ≥ 3. Error bars: + SEM. (e) Pt induced a concentration- dependent increase in mitochondrial mass after 48 h. FACS measurements of NAO fluorescence in HeLa cells. Mean values + SEM; *N* ≥ 4.

Since ROS and Ca^2+^ are well-known inducers of the mitochondrial permeability transition (MPT), we looked also at the effects of Pt on mitochondrial membrane potential. 25 *μ*M Pt induced a rapid loss of transmembrane potential. 10 *μ*M Pt had a milder, and 1 *μ*M only a slight (if any), effect (Figure 5(a)). We concentrated on 10 *μ*M Pt, a physiologically relevant concentration with a more moderate impact. That the MPT is responsible, at least in part, for depolarization is confirmed by the partial protective effect of NIM811 (Figure 5(b), curve iii). As expected, the combined action of BAPTA-AM and NIM811 blocked mitochondrial depolarization (Figure 5(b), curve iv). However even in their presence there was a clear – albeit reduced – Pt-induced nuclear migration of TFEB-GFP (Figure 5(c)). Mitochondrial damage may thus contribute in a dose-dependent manner to Pt-elicited TFEB relocation, but it is not *the* crucial factor.

We hypothesized Pt might also activate mitogenesis, the “symmetrical” process mirroring TFEB-regulated mitophagy [87, 88]. Accordingly, the transcript of *PPARGC1a* increased in HeLa cells treated with Pt (Figure 5(d)). Correspondingly, the mitochondrial content, evaluated using NAO staining [89] and FACS (Figure 5(e)), also increased.

### 3.8. *In Vivo* Effects

#### 3.8.1. Zebrafish Model of ColVI Deficiency

As a first step to verify the relevance of these findings *in vivo,* we turned to zebrafish (*Danio rerio*) a versatile model system which has already been widely used in autophagy studies (e.g. [90–94]).

We tested Pt in a zebrafish embryo model of Collagen VI-dependent muscular dystrophy [71]. In mammals, loss of this extracellular matrix component results in repression of autophagy and mitochondrial dysfunction, causing a spectrum of muscle dystrophies [12, 13, 95]. Administration of spermidine, an autophagy inducer, has been observed to lead to improvements in a dystrophic mouse model [96].

**Figure 6:**
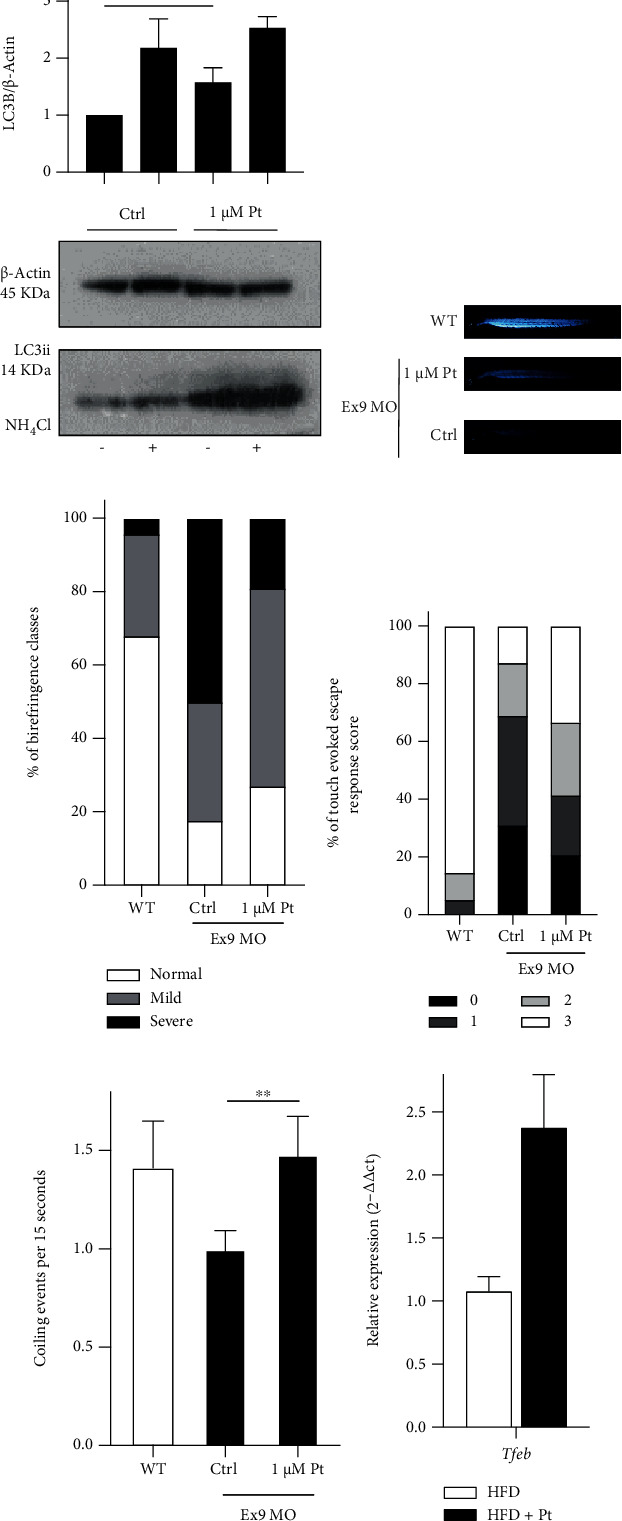
Pt-induced autophagy and TFEB activation *in vivo*. (a–e) Evidence of Pt-induced autophagy and functional improvement in dystrophic zebrafish morphants with altered Collagen VI. (a) Representative Western blot and quantification of the lipidated form of LC3 in zebrafish embryos treated as indicated. Mean values + SEM; *N* ≥ 3. (b, c) Birefringence assay. (b) Representative images from WT fish or ColVI morphants (Ex9 MO) after treatment with Pt or 0.1% DMSO (Ctrl). (c) 1 *μ*M Pt induced a significant increase in the birefringence of morphants at 48 hpf. The percentage of fish showing a severe phenotype of ColVI-related myopathy was considerably reduced by 1 *μ*M Pt (Pt vs Ctrl: *p* = 0.016). Mean values + SEM. *N* ≥ 60 embryos for each condition, from at least 3 separate experiments. (d) Touch-evoked response assay. 1 *μ*M Pt increased the percentage of fish showing mild or normal phenotypes (*p* = 0.0007). Responses were evoked by touching 48 hpf embryos with a tip. *N* ≥ 70 events for each condition, from at least 3 separate experiments. (e) Spontaneous coiling events of ColVI morphants measured at 24 hpf after 3 hours of treatment with Pt. 1 *μ*M Pt completely rescues the deficit of spontaneous movements. Mean values + SEM. *N* ≥ 100 events for each condition, from at least 3 separate experiments. (f) RT-qPCR of Tfeb transcription in inguinal white adipose tissues from obese (HFD) and Pt-treated (HFD + Pt) mice. Mean values + SEM, *N* ≥ 14 mice for each condition.

1 *μ*M Pt induced significant increase of the lipidation levels of LC3, the autophagy marker (Figure 6(a)), and a recovery of the muscle structure (as assessed by the birefringence assay) (Figures 6(b) and 6(c)). An improvement of motility was also observed by measuring the touch-evoked escape response (Figure 6(d)) and spontaneous coiling events (Figure 6(e)).

#### 3.8.2. Mouse Model of Diet-Induced Obesity

We have recently reported that long-term Pt administration to mice fed a high-fat diet limited weight gain and promoted “browning” of inguinal white adipose tissue (iWAT) [64].

RT-qPCR analysis of iWAT from HFD and HFD + Pt mice showed that Pt administration increases the expression, among other genes, of *Ppargc1a* [64], coherently with the observations made with HeLa cells (Figure 5(d)). Since TFEB controls the expression of its own gene [97] we assessed here the effects of Pt supplementation on the expression of *Tfeb*. Indeed, transcription of the *Tfeb* gene was enhanced in the iWAT of obese animals treated with Pt (Figure 6(f)).

## 4. Discussion

The results presented in this paper show that Pt in the *μ*M range may induce TFEB activation both *in vitro* and *in vivo.*

First, we focused on the mechanisms contributing to the Pt-induced migration of TFEB to the nucleus. We used HeLa cells, a well-known and characterized cell line; the same cells stably transfected with a TFEB-GFP chimera were also available, allowing us to directly follow the subcellular localization of TFEB through fluorescence microscopy.

The concentrations of Pt we used in our experiments (in the 1-25 *μ*M range) can be reached without difficulty in several organs of rodents receiving oral doses of Pt of about 20 mg/Kg body weight [56], and even more easily if a prodrug comprising an amino-acid is used instead of the compound as such [98]. In these key respects, the performance of Pt is superior to that of more celebrated resveratrol.

Pt induced a concentration-dependent migration of TFEB to the cell nucleus and an increase in the transcription levels of a set of TFEB target genes (Figure 1). The signaling cascades leading to this relocation involve generation of superoxide, attributed to the interaction of Pt with complex I of the mitochondrial respiratory chain (Figure 2). These findings are coherent with the well-known involvement of ROS (and mitochondrial ROS in particular) in the regulation/activation of autophagy [74–77], and with our previous data reporting that a mitochondria-targeted derivative of Pt accumulates in the organelles, interacts with respiratory chain complexes, and determines mitochondrial production of ROS [70]. As recently reported by Wang et al., 2019 [76], ROS can prompt TFEB migration through direct oxidation of a specific Cysteine residue. Also other proteins involved in signaling via TFEB can be directly activated by ROS, for example the phosphatase PPA2, which dephosphorylates TFEB and TFE3 promoting their migration to the nucleus [99]. Another potentially important redox-sensitive component of the autophagy-inducing signaling network, the kinase LKB1, is not present in HeLa cells [100].

Other important players can be involved in the regulation of TFEB migration, depending on circumstances (Suppl. Figure 1 and references provided there). In particular, mTORC1 and calcium/calcineurin have been shown to be key actors in nutrient deprivation-induced TFEB migration and autophagy onset [27, 86, 101]. Indeed, we observed that Pt determines mTORC1 inhibition (Figure 3), and the release of Ca^2+^ from intracellular stores (Figure 4), thus partially overlapping the signaling cascades relevant in the nutrient deprivation model. Interestingly, these pathways may also be influenced by ROS (Suppl. Figure 1).

The increased phosphorylation of AMPK (Figure 3(c)) and of CREB (Suppl. Figure 4D, 4E) we observed may be ascribed to Ca^2+^ signaling: both AMPK [102] and CREB [103] can in fact be activated via phosphorylation by Ca^2+^-dependent CaM Kinases (CaMKKs), in particular CaMKK*β* (as schematized in Suppl. Figure 1). AMPK in turn can phosphorylate and inhibit mTORC1, contributing to TFEB migration to the nucleus. Additionally, it is worth to mention that mTORC1 might be also directly inhibited, as reported for resveratrol [39].

Pt also weakly inhibits phosphodiesterase activity in HeLa cell lysates, but this is not sufficient to determine an increase of cAMP levels in this specific experimental system.

Cytosolic Ca^2+^ levels, and their modulation downstream of ER Ca^2+^ content, have been proposed to be of key importance for the autophagic process in other studies as well [45, 104]. In starvation-induced autophagy the lysosomal Ca^2+^ (and Fe^2+^) channel TRPML1/MCOLN1 is the ROS-sensitive element linking ROS and Ca^2+^ signaling [105]. Other possibly relevant redox-regulated intracellular ion channels and “pumps” include ROS-activated IP_3_R, RyR and MCU, and the ROS-inhibited SERCA pumps of the ER. Treatment of HeLa cells with H_2_O_2_ induced release of Ca^2+^ from intracellular stores to the cytosol (not shown), confirming the relationship between ROS and Ca^2+^ levels (e.g. [106]).

At relatively high – but still pharmacologically achievable *in vivo* in some organs such as the liver - Pt concentrations (25 *μ*M), Ca^2+^ is rapidly and massively released from the ER/mitochondria stores (Figures 4(a) and 4(b)). The characteristics of the Ca^2+^ “wave” warrant the conclusion that Ca^2+^ may largely have its origin in the ER. Indeed, previous depletion of ER Ca^2+^ eliminates the Pt-induced transient (Figure 4(c)). The mitochondria are however also involved (Figure 4(b)), and the observations suggest that ER Ca^2+^ may be transferred to mitochondria via contact sites between the organelles or, in some cases, via the cytosol. The mitochondria in turn release it to the cytosol in a process involving the mitochondrial permeability transition (MPT), a phenomenon well known to be induced by ROS and matrix Ca^2+^ overload, which leads to depolarization and massive release of Ca^2+^ from the organelles [107]. Indeed, the complexity of the Pt-elicited cytosolic Ca^2+^ signal, and the effect of NIM811, an MPT inhibitor, on the Ca^2+^ transient (Figure 4(b), curve iv) suggested MPT occurrence. A consequence of mitochondrial damage is TFEB migration to the nucleus [108]. However, even in the presence of MPT inhibitors we observed a clear Pt-induced TFEB migration. The result is a marked but transient increase of cytosolic Ca^2+^, which then returns to approximately basal level. At lower Pt concentrations (10 *μ*M) the characteristics of the cytosolic Ca^2+^ signal are different: the increase is much less marked and slower, and leads to a long-lasting elevation (Figure 4(e)). Under these circumstances mitochondrial depolarization still takes place, although it is less marked (Figure 5(a)). Such an “uncoupling” effect, in addition to contributing to the onset of autophagy, may partly explain the slimming effects of Pt supplementation in a murine model of obese HFD-fed mice [64].

In addition to inducing MPT, Pt also induced mitogenesis. The simultaneous and balanced occurrence of both processes would guarantee a renewal of mitochondria in the cell, replacing malfunctioning organelles with newly generated ones, a process of great pathophysiological relevance [109, 110]. AMPK, which is activated by Pt (see above), plays an important role in mitochondrial biogenesis via upregulation of PGC-1*α*/*Ppargc1a* [111, 112]. *Ppargc1a* expression is also directly enhanced by TFEB [97, 113, 114] and it may be upregulated also by CREB [115], which we have observed to be phosphorylated in our system, and by other pathways downstream of CaMK and calcineurin [116, 117].

Pt-induced effects were finally confirmed in *in vivo* models. Autophagy-mediated beneficial effects of Pt on Collagen VI-dependent dystrophies were assessed here on a zebrafish model of ColVI deficiency, and were recently demonstrated also in a mouse model of this pathology [118], further underscoring the therapeutic potential of this natural compound.

We also observed an increased transcription of *Ppargc1a* and *Tfeb* genes in the inguinal white adipose tissue of a mouse model of diet-induced obesity. Autophagy has emerged as a counteracting mechanism opposing obesity and obesity-associated metabolic syndrome/type II diabetes [66, 119–121]. TFEB acts as the master regulator also in this case [114] and its activation is a therapeutic goal.

## 5. Conclusions and Perspectives

Multiple mechanisms are activated by Pt and contribute to TFEB activation. This confers Pt the potential to function as a health-improving agent in all conditions which would benefit from the stimulation of autophagy, lysosomal biogenesis and/or mitochondrial turnover. These include muscular dystrophies: in addition to ColVI-related myopathies, it may be predicted that favorable effects would be obtained also in models of Duchenne's and possibly other muscular dystrophies [122]. *Tfeb* and *Ppargc1a* transcription are enhanced by Pt supplementation in the inguinal white adipose tissue of diet-induced obese mice and may contribute to the reduced weight gain observed in Pt-consuming mice [64]. Another relevant and promising field of application may be neurodegenerative and aging-related disorders. This is in view of the recognized beneficial role of autophagy and CREB activation in these pathologies and also because *μ*M concentrations of unmodified Pt are reached in the brain after oral administration (at least in the rat) [56]. Indeed, it has been already reported that administration of Pt leads to improvements of cognitive performance [59, 81, 123]. A widespread inclusion of this natural molecule as an additive in functional foods deserves consideration, as it may contribute significantly to public health. Dosage must however be carefully controlled to avoid the possibility of side effects emerging at high Pt concentrations.

## Data Availability

The data that support the findings of this study are available from the corresponding authors upon request.

## Abbreviations

BAPTA-AM: 1,2-Bis(2-aminophenoxy)ethane-N,N,N′,N′-tetraacetic acid tetrakis (acetoxymethyl ester)
BCA: Bicinchoninic acid
BSA: Bovine serum albumin
CPA: Cyclopiazonic acid
ColVI: Collagen VI
CsA: Cyclosporine A
CsH: Cyclosporine H
DMEM: Dulbecco's Modified Eagle Medium
DTT: Dithiothreitol
ECM: Extracellular matrix
EGTA: Ethylene glycol-bis(2-aminoethylether)-N,N,N′,N′-tetraacetic acid
ER: Endoplasmic reticulum
FBS: Fetal bovine serum
FCCP: Carbonyl cyanide 4-(trifluoromethoxy)phenylhydrazone
GFP: Green fluorescent protein
HBSS: Hanks' Balanced Salt solution
HFD: High-fat diet
Hpf: Hours postfertilization
IBMX: 3-Isobutyl-1-methylxanthine
MLS-A1: 2-[2-(3,4-Dihydro-2,2,4-trimethyl-1(2H)-quinolinyl)-2-oxoethyl]-1H-isoindole-1,3(2H)-dione
MPT: Mitochondrial permeability transition
NAC: N-Acetyl-L-cysteine
NAO: Acridine Orange 10-nonyl bromide
NIM811: N-Methyl-4-isoleucine cyclosporin
PDE: Phosphodiesterase
PBS: Phosphate-buffered saline
PMSF: Phenylmethylsulfonyl fluoride
Pt: Pterostilbene
PVDF: Polyvinylidene difluoride
TBST: Tris-buffered saline containing tween 0.1%
TFEB: Transcription factor EB
RLM: Rat liver mitochondria
ROI: Region of interest
ROS: Reactive oxygen species
SDS: Sodium dodecyl sulfate
TMRM: Tetramethylrhodamine methyl ester
WT: Wild-type.

## Supplementary Materials

Supplementary Materials Supplementary Table 1: list of forward and reverse primers used for RT-qPCR analysis. Supplementary Figure 1: signaling pathways involved in TFEB activation. Supplementary Figure 2: Pt triggers autophagy in cultured HeLa cells. Supplementary Figure 3: representative images of WT HeLa cells loaded with MitoSOX™ dye and treated as indicated. Supplementary Figure 4: modulation of cAMP levels is not a key element in Pt-induced AMPK phosphorylation and TFEB migration. Supplementary Figure 5: role of lysosomal Ca^2+^ in TFEB migration.

## References

[1] Swart C., Du Toit A., Loos B. (2016). Autophagy and the invisible line between life and death. *European Journal of Cell Biology*.

[2] Mariño G., Niso-Santano M., Baehrecke E. H., Kroemer G. (2014). Self-consumption: the interplay of autophagy and apoptosis. *Nature Reviews Molecular Cell Biology*.

[3] Nagata R., Nakamura M., Sanaki Y., Igaki T. (2019). Cell competition is driven by autophagy. *Developmental Cell*.

[4] Thorburn A. (2018). Thematic minireview: Autophagy and disease. *The Journal of Biological Chemistry*.

[5] Khandia R., Dadar M., Munjal A. (2019). A comprehensive review of autophagy and its various roles in infectious, non-infectious, and lifestyle diseases: current knowledge and prospects for disease prevention, novel drug design, and therapy. *Cell*.

[6] Saha S., Panigrahi D. P., Patil S., Bhutia S. K. (2018). Autophagy in health and disease: a comprehensive review. *Biomedicine & Pharmacotherapy*.

[7] Klionsky D. J., Petroni G., Amaravadi R. K. (2021). Autophagy in major human diseases. *The EMBO Journal*.

[8] Kitada M., Koya D. (2021). Autophagy in metabolic disease and ageing. *Nature Reviews Endocrinology*.

[9] Ariosa A. R., Lahiri V., Lei Y. (2021). A perspective on the role of autophagy in cancer. *Biochimica et Biophysica Acta - Molecular Basis of Disease*.

[10] Zheng X., Li S., Li J. (2020). Hexavalent chromium induces renal apoptosis and autophagy via disordering the balance of mitochondrial dynamics in rats. *Ecotoxicology and Environmental Safety*.

[11] Namkoong S., Cho C. S., Semple I., Lee J. H. (2018). Autophagy dysregulation and obesity-associated pathologies. *Molecules and Cells*.

[12] Castagnaro S., Chrisam M., Cescon M., Braghetta P., Grumati P., Bonaldo P. (2018). Extracellular collagen VI has Prosurvival and autophagy instructive properties in mouse fibroblasts. *Frontiers in Physiology*.

[13] Castagnaro S., Gambarotto L., Cescon M., Bonaldo P. (2021). Autophagy in the mesh of collagen VI. *Matrix Biology*.

[14] Schaefer L., Dikic I. (2021). Autophagy: instructions from the extracellular matrix. *Matrix Biology*.

[15] Neill T., Kapoor A., Xie C., Buraschi S., Iozzo R. V. (2021). A functional outside-in signaling network of proteoglycans and matrix molecules regulating autophagy. *Matrix Biology*.

[16] Yang D., Yang Q., Fu N. (2021). Hexavalent chromium induced heart dysfunction via Sesn2-mediated impairment of mitochondrial function and energy supply. *Chemosphere*.

[17] Lv Y., Jiang H., Li S. (2020). Sulforaphane prevents chromium-induced lung injury in rats via activation of the Akt/GSK-3*β*/Fyn pathway. *Environmental Pollution*.

[18] Narendra D. P. (2021). Managing risky assets - mitophagy in vivo. *Journal of Cell Science*.

[19] Li S., Zhang J., Liu C. (2021). The role of Mitophagy in regulating cell death. *Oxidative Medicine and Cellular Longevity*.

[20] Luan Y., Luan Y., Feng Q., Chen X., Ren K. D., Yang Y. (2021). Emerging role of Mitophagy in the heart: therapeutic potentials to modulate Mitophagy in cardiac diseases. *Oxidative Medicine and Cellular Longevity*.

[21] Redza-Dutordoir M., Averill-Bates D. A. (2021). Interactions between reactive oxygen species and autophagy: special issue: death mechanisms in cellular homeostasis. *Biochimica et Biophysica Acta (BBA) - Molecular Cell Research*.

[22] Li D., Ding Z., Du K., Ye X., Cheng S. (2021). Reactive oxygen species as a link between antioxidant pathways and autophagy. *Oxidative Medicine and Cellular Longevity*.

[23] Sardiello M., Palmieri M., di Ronza A. (2009). A gene network regulating lysosomal biogenesis and function. *Science*.

[24] Settembre C., di Malta C., Polito V. A. (2011). TFEB links autophagy to lysosomal biogenesis. *Science*.

[25] Napolitano G., Ballabio A. (2016). TFEB at a glance. *Journal of Cell Science*.

[26] Di Malta C., Cinque L., Settembre C. (2019). Transcriptional regulation of autophagy: mechanisms and diseases. *Frontiers in Cell and Development Biology*.

[27] Puertollano R., Ferguson S. M., Brugarolas J., Ballabio A. (2018). The complex relationship between TFEB transcription factor phosphorylation and subcellular localization. *The EMBO Journal*.

[28] La Spina M., Contreras P. S., Rissone A., Meena N. K., Jeong E., Martina J. A. (2021). MiT/TFE family of transcription factors: an evolutionary perspective. *Frontiers in Cell and Development Biology*.

[29] Martina J. A., Diab H. I., Lishu L. (2014). The nutrient-responsive transcription factor TFE3 promotes autophagy, lysosomal biogenesis, and clearance of cellular debris. *Science Signaling*.

[30] Thellung S., Corsaro A., Nizzari M., Barbieri F., Florio T. (2019). Autophagy activator drugs: a new opportunity in neuroprotection from misfolded protein toxicity. *International Journal of Molecular Sciences*.

[31] Heras-Sandoval D., Perez-Rojas J. M., Pedraza-Chaverri J. (2020). Novel compounds for the modulation of mTOR and autophagy to treat neurodegenerative diseases. *Cellular Signalling*.

[32] Cuomo F., Altucci L., Cobellis G. (2019). Autophagy Function and Dysfunction: Potential Drugs as Anti-Cancer Therapy. *Cancers*.

[33] Wang C., Hu Q., Shen H. M. (2016). Pharmacological inhibitors of autophagy as novel cancer therapeutic agents. *Pharmacological Research*.

[34] Ren J., Zhang Y. (2018). Targeting autophagy in aging and aging-related cardiovascular diseases. *Trends in Pharmacological Sciences*.

[35] Yin X., Xin H., Mao S., Wu G., Guo L. (2019). The role of autophagy in sepsis: protection and injury to organs. *Frontiers in Physiology*.

[36] Levy J. M. M., Towers C. G., Thorburn A. (2017). Targeting autophagy in cancer. *Nature Reviews Cancer*.

[37] Hajieva P. (2017). The effect of polyphenols on protein degradation pathways: implications for neuroprotection. *Molecules*.

[38] Chen M. L., Yi L., Jin X. (2013). Resveratrol attenuates vascular endothelial inflammation by inducing autophagy through the cAMP signaling pathway. *Autophagy*.

[39] Park D., Jeong H., Lee M. N. (2016). Resveratrol induces autophagy by directly inhibiting mTOR through ATP competition. *Scientific Reports*.

[40] Xie Q., Chen Y., Tan H., Liu B., Zheng L. L., Mu Y. (2021). Targeting autophagy with natural compounds in cancer: a renewed perspective from molecular mechanisms to targeted therapy. *Frontiers in Pharmacology*.

[41] Miguel C. A., Noya-Riobó M. V., Mazzone G. L., Villar M. J., Coronel M. F. (2021). Antioxidant, anti-inflammatory and neuroprotective actions of resveratrol after experimental nervous system insults. Special focus on the molecular mechanisms involved. *Neurochemistry International*.

[42] Ma R., Yu D., Peng Y. (2021). Resveratrol induces AMPK and mTOR signaling inhibition-mediated autophagy and apoptosis in multiple myeloma cells. *Acta Biochimica et Biophysica Sinica*.

[43] Josifovska N., Albert R., Nagymihály R. (2020). Resveratrol as inducer of autophagy, pro-survival, and anti-inflammatory stimuli in cultured human RPE cells. *International Journal of Molecular Sciences*.

[44] Li S., Zheng X., Zhang X. (2021). Exploring the liver fibrosis induced by deltamethrin exposure in quails and elucidating the protective mechanism of resveratrol. *Ecotoxicology and Environmental Safety*.

[45] Luyten T., Welkenhuyzen K., Roest G. (2017). Resveratrol-induced autophagy is dependent on IP_3_Rs and on cytosolic Ca^2 +^. *Biochimica et Biophysica Acta*.

[46] Manach C., Williamson G., Morand C., Scalbert A., Rémésy C. (2005). Bioavailability and bioefficacy of polyphenols in humans. I. Review of 97 bioavailability studies. *The American Journal of Clinical Nutrition*.

[47] Wenzel E., Somoza V. (2005). Metabolism and bioavailability of trans-resveratrol. *Molecular Nutrition & Food Research*.

[48] Walle T. (2011). Bioavailability of resveratrol. *Annals of the New York Academy of Sciences*.

[49] Estrela J. M., Ortega A., Mena S., Rodriguez M. L., Asensi M. (2013). Pterostilbene: Biomedical applications. *Critical Reviews in Clinical Laboratory Sciences*.

[50] McCormack D., McFadden D. (2012). Pterostilbene and cancer: current review. *The Journal of Surgical Research*.

[51] McCormack D., McFadden D. (2013). A review of pterostilbene antioxidant activity and disease modification. *Oxidative Medicine and Cellular Longevity*.

[52] Lin W. S., Leland J. V., Ho C. T., Pan M. H. (2020). Occurrence, Bioavailability, Anti-inflammatory, and Anticancer Effects of Pterostilbene. *Journal of Agricultural and Food Chemistry*.

[53] Lin H. S., Yue B. D., Ho P. C. (2009). Determination of pterostilbene in rat plasma by a simple HPLC-UV method and its application in pre-clinical pharmacokinetic study. *Biomedical Chromatography*.

[54] Kapetanovic I. M., Muzzio M., Huang Z., Thompson T. N., McCormick D. L. (2011). Pharmacokinetics, oral bioavailability, and metabolic profile of resveratrol and its dimethylether analog, pterostilbene, in rats. *Cancer Chemotherapy and Pharmacology*.

[55] Yeo S. C., Ho P. C., Lin H. S. (2013). Pharmacokinetics of pterostilbene in Sprague-Dawley rats: the impacts of aqueous solubility, fasting, dose escalation, and dosing route on bioavailability. *Molecular Nutrition & Food Research*.

[56] Azzolini M., la Spina M., Mattarei A., Paradisi C., Zoratti M., Biasutto L. (2014). Pharmacokinetics and tissue distribution of pterostilbene in the rat. *Molecular Nutrition & Food Research*.

[57] Nutakul W., Sobers H. S., Qiu P. (2011). Inhibitory effects of resveratrol and pterostilbene on human colon cancer cells: a side-by-side comparison. *Journal of Agricultural and Food Chemistry*.

[58] Chiou Y. S., Tsai M. L., Nagabhushanam K. (2011). Pterostilbene is more potent than resveratrol in preventing azoxymethane (AOM)-induced colon tumorigenesis via activation of the NF-E2-related factor 2 (Nrf2)-mediated antioxidant signaling pathway. *Journal of Agricultural and Food Chemistry*.

[59] Chang J., Rimando A., Pallas M. (2012). Low-dose pterostilbene, but not resveratrol, is a potent neuromodulator in aging and Alzheimer's disease. *Neurobiology of Aging*.

[60] Chakraborty A., Bodipati N., Demonacos M. K., Peddinti R., Ghosh K., Roy P. (2012). Long term induction by pterostilbene results in autophagy and cellular differentiation in MCF-7 cells via ROS dependent pathway. *Molecular and Cellular Endocrinology*.

[61] Mena S., Rodríguez M. L., Ponsoda X., Estrela J. M., Jäättela M., Ortega A. L. (2012). Pterostilbene-induced tumor cytotoxicity: a lysosomal membrane permeabilization-dependent mechanism. *PLoS One*.

[62] Siedlecka-Kroplewska K., Jozwik A., Boguslawski W. (2013). Pterostilbene induces accumulation of autophagic vacuoles followed by cell death in HL60 human leukemia cells. *Journal of Physiology and Pharmacology*.

[63] Ko C. P., Lin C. W., Chen M. K., Yang S. F., Chiou H. L., Hsieh M. J. (2015). Pterostilbene induce autophagy on human oral cancer cells through modulation of Akt and mitogen-activated protein kinase pathway. *Oral Oncology*.

[64] la Spina M., Galletta E., Azzolini M. (2019). Browning effects of a chronic Pterostilbene supplementation in mice fed a high-fat diet. *International Journal of Molecular Sciences*.

[65] Grumati P., Coletto L., Sabatelli P. (2010). Autophagy is defective in collagen VI muscular dystrophies, and its reactivation rescues myofiber degeneration. *Nature Medicine*.

[66] Sarparanta J., Garcia-Macia M., Singh R. (2017). Autophagy and mitochondria in obesity and type 2 diabetes. *Current Diabetes Reviews*.

[67] Settembre C., Zoncu R., Medina D. L. (2012). A lysosome-to-nucleus signalling mechanism senses and regulates the lysosome via mTOR and TFEB. *The EMBO Journal*.

[68] Di Virgilio F., Steinberg T. H., Silverstein S. C. (1990). Inhibition of Fura-2 sequestration and secretion with organic anion transport blockers. *Cell Calcium*.

[69] Livak K. J., Schmittgen T. D. (2001). Analysis of Relative Gene Expression Data Using Real-Time Quantitative PCR and the 2^−*ΔΔ* _C_^_T_ Method. *Methods*.

[70] Sassi N., Mattarei A., Azzolini M. (2014). Cytotoxicity of mitochondria-targeted resveratrol derivatives: interactions with respiratory chain complexes and ATP synthase. *Biochimica et Biophysica Acta*.

[71] Telfer W. R., Busta A. S., Bonnemann C. G., Feldman E. L., Dowling J. J. (2010). Zebrafish models of collagen VI-related myopathies. *Human Molecular Genetics*.

[72] Berger J., Sztal T., Currie P. D. (2012). Quantification of birefringence readily measures the level of muscle damage in zebrafish. *Biochemical and Biophysical Research Communications*.

[73] Palmieri M., Impey S., Kang H. (2011). Characterization of the CLEAR network reveals an integrated control of cellular clearance pathways. *Human Molecular Genetics*.

[74] Chen Y., Gibson S. B. (2008). Is mitochondrial generation of reactive oxygen species a trigger for autophagy?. *Autophagy*.

[75] Zhang L., Wang K., Lei Y., Li Q., Nice E. C., Huang C. (2015). Redox signaling: potential arbitrator of autophagy and apoptosis in therapeutic response. *Free Radical Biology & Medicine*.

[76] Wang H., Wang N., Xu D. (2020). Oxidation of multiple MiT/TFE transcription factors links oxidative stress to transcriptional control of autophagy and lysosome biogenesis. *Autophagy*.

[77] Roca-Agujetas V., de Dios C., Lestón L., Marí M., Morales A., Colell A. (2019). Recent insights into the mitochondrial role in autophagy and its regulation by oxidative stress. *Oxidative Medicine and Cellular Longevity*.

[78] Roelofs B. A., Ge S. X., Studlack P. E., Polster B. M. (2015). Low micromolar concentrations of the superoxide probe MitoSOX uncouple neural mitochondria and inhibit complex IV. *Free Radical Biology & Medicine*.

[79] Göransson O., McBride A., Hawley S. A. (2007). Mechanism of Action of A-769662, a Valuable Tool for Activation of AMP- activated Protein Kinase∗. *The Journal of Biological Chemistry*.

[80] Park S. J., Ahmad F., Philp A. (2012). Resveratrol ameliorates aging-related metabolic phenotypes by inhibiting cAMP phosphodiesterases. *Cell*.

[81] la Spina M., Sansevero G., Biasutto L. (2019). Pterostilbene improves cognitive performance in aged rats: an in vivo study. *Cellular Physiology and Biochemistry*.

[82] Vallese F., Barazzuol L., Maso L., Brini M., Calì T. (2020). ER-mitochondria calcium transfer, organelle contacts and neurodegenerative diseases. *Advances in Experimental Medicine and Biology*.

[83] Rossi A., Pizzo P., Filadi R. (2019). Calcium, mitochondria and cell metabolism: a functional triangle in bioenergetics. *Biochimica et Biophysica Acta (BBA) - Molecular Cell Research*.

[84] Veeresh P., Kaur H., Sarmah D. (2019). Endoplasmic reticulum-mitochondria crosstalk: from junction to function across neurological disorders. *Annals of the New York Academy of Sciences*.

[85] Wu H., Carvalho P., Voeltz G. K. (2018). Here, there, and everywhere: the importance of ER membrane contact sites. *Science*.

[86] Medina D. L., di Paola S., Peluso I. (2015). Lysosomal calcium signalling regulates autophagy through calcineurin and TFEB. *Nature Cell Biology*.

[87] Ivankovic D., Chau K. Y., Schapira A. H. V., Gegg M. E. (2016). Mitochondrial and lysosomal biogenesis are activated following PINK1/parkin- mediated mitophagy. *Journal of Neurochemistry*.

[88] Siddiqui A., Bhaumik D., Chinta S. J. (2015). Mitochondrial quality control via the PGC1 -TFEB signaling pathway is compromised by Parkin Q311X mutation but independently restored by rapamycin. *The Journal of Neuroscience*.

[89] Benel L., Ronot X., Mounolou J. C., Gaudemer F., Adolphe M. (1989). Compared flow cytometric analysis of mitochondria using 10-n-nonyl Acridine Orange and rhodamine 123. *Basic and Applied Histochemistry*.

[90] Lopez A., Lee S. E., Wojta K. (2017). A152T tau allele causes neurodegeneration that can be ameliorated in a zebrafish model by autophagy induction. *Brain*.

[91] Zhang Y., Nguyen D. T., Olzomer E. M. (2017). Rescue of Pink1 deficiency by stress-dependent activation of autophagy. *Cell Chemical Biology*.

[92] Fodor E., Sigmond T., Ari E. (2017). Methods to study autophagy in zebrafish. *Methods in Enzymology*.

[93] Zhu J., Xia R., Liu Z. (2020). Fenvalerate triggers Parkinson-like symptom during zebrafish development through initiation of autophagy and p38 MAPK/mTOR signaling pathway. *Chemosphere*.

[94] Khuansuwan S., Barnhill L. M., Cheng S., Bronstein J. M. (2019). A novel transgenic zebrafish line allows for in vivo quantification of autophagic activity in neurons. *Autophagy*.

[95] Lamandé S. R., Bateman J. F. (2018). Collagen VI disorders: insights on form and function in the extracellular matrix and beyond. *Matrix Biology*.

[96] Chrisam M., Pirozzi M., Castagnaro S. (2015). Reactivation of autophagy by spermidine ameliorates the myopathic defects of collagen VI-null mice. *Autophagy*.

[97] Settembre C., de Cegli R., Mansueto G. (2013). TFEB controls cellular lipid metabolism through a starvation-induced autoregulatory loop. *Nature Cell Biology*.

[98] Azzolini M., Mattarei A., la Spina M. (2017). New natural amino acid-bearing prodrugs boost pterostilbene's oral pharmacokinetic and distribution profile. *European Journal of Pharmaceutics and Biopharmaceutics*.

[99] Martina J. A., Puertollano R. (2018). Protein phosphatase 2A stimulates activation of TFEB and TFE3 transcription factors in response to oxidative stress. *The Journal of Biological Chemistry*.

[100] Zhang X., Chen H., Wang X., Zhao W., Chen J. J. (2014). Expression and transcriptional profiling of the LKB1 tumor suppressor in cervical cancer cells. *Gynecologic Oncology*.

[101] Napolitano G., Esposito A., Choi H. (2018). mTOR-dependent phosphorylation controls TFEB nuclear export. *Nature Communications*.

[102] Woods A., Dickerson K., Heath R. (2005). Ca^2+^/calmodulin-dependent protein kinase kinase-*β* acts upstream of AMP-activated protein kinase in mammalian cells. *Cell Metabolism*.

[103] Yan X., Liu J., Ye Z. (2016). CaMKII-mediated CREB phosphorylation is involved in Ca2+-induced BDNF mRNA transcription and neurite outgrowth promoted by electrical stimulation. *PLoS One*.

[104] Fedeli C., Filadi R., Rossi A., Mammucari C., Pizzo P. (2019). PSEN2 (presenilin 2) mutants linked to familial Alzheimer disease impair autophagy by altering Ca2+homeostasis. *Autophagy*.

[105] Zhang X., Cheng X., Yu L. (2016). MCOLN1 is a ROS sensor in lysosomes that regulates autophagy. *Nature Communications*.

[106] Volk T., Hensel M., Kox W. J. (1997). Transient Ca2+ changes in endothelial cells induced by low doses of reactive oxygen species: role of hydrogen peroxide. *Molecular and Cellular Biochemistry*.

[107] Zoratti M., de Marchi U., Biasutto L., Szabò I. (2010). Electrophysiology clarifies the megariddles of the mitochondrial permeability transition pore. *FEBS Letters*.

[108] Nezich C. L., Wang C., Fogel A. I., Youle R. J. (2015). MiT/TFE transcription factors are activated during mitophagy downstream of Parkin and Atg5. *The Journal of Cell Biology*.

[109] Ploumi C., Daskalaki I., Tavernarakis N. (2017). Mitochondrial biogenesis and clearance: a balancing act. *The FEBS Journal*.

[110] Palikaras K., Lionaki E., Tavernarakis N. (2015). Coupling mitogenesis and mitophagy for longevity. *Autophagy*.

[111] Uittenbogaard M., Chiaramello A. (2014). Mitochondrial biogenesis: a therapeutic target for neurodevelopmental disorders and neurodegenerative diseases. *Current Pharmaceutical Design*.

[112] Marin T. L., Gongol B., Zhang F. (2017). AMPK promotes mitochondrial biogenesis and function by phosphorylating the epigenetic factors DNMT1, RBBP7, and HAT1. *Science Signaling*.

[113] Mansueto G., Armani A., Viscomi C. (2017). Transcription factor EB controls metabolic flexibility during exercise. *Cell Metabolism*.

[114] Evans T. D., Zhang X., Jeong S. J. (2019). TFEB drives PGC-1*α* expression in adipocytes to protect against diet-induced metabolic dysfunction. *Science Signaling*.

[115] Herzig S., Long F., Jhala U. S. (2001). CREB regulates hepatic gluconeogenesis through the coactivator PGC-1. *Nature*.

[116] Hood D. A., Tryon L. D., Carter H. N., Kim Y., Chen C. C. W. (2016). Unravelling the mechanisms regulating muscle mitochondrial biogenesis. *The Biochemical Journal*.

[117] Jornayvaz F. R., Shulman G. I. (2010). Regulation of mitochondrial biogenesis. *Essays in Biochemistry*.

[118] Metti S., Gambarotto L., Chrisam M. (2020). The polyphenol Pterostilbene ameliorates the Myopathic phenotype of collagen VI deficient mice via autophagy induction. *Frontiers in Cell and Development Biology*.

[119] Khawar M. B., Gao H., Li W. (2019). Autophagy and lipid metabolism. *Advances in Experimental Medicine and Biology*.

[120] Su Z., Nie Y., Huang X. (2019). Mitophagy in hepatic insulin resistance: therapeutic potential and concerns. *Frontiers in Pharmacology*.

[121] Lee Y. H., Kim J., Park K., Lee M. S. (2019). *β*-cell autophagy: Mechanism and role in *β*-cell dysfunction. *Molecular Metabolism*.

[122] Sandri M., Coletto L., Grumati P., Bonaldo P. (2013). Misregulation of autophagy and protein degradation systems in myopathies and muscular dystrophies. *Journal of Cell Science*.

[123] Poulose S. M., Thangthaeng N., Miller M. G., Shukitt-Hale B. (2015). Effects of pterostilbene and resveratrol on brain and behavior. *Neurochemistry International*.

[124] La Spina M. (2017). *Pharmacology, biochemistry and biomedical applications of plant stilbenes, [Ph.D. thesis]*.

