# Supplementary material for: Multiple Mechanisms Converging on Transcription Factor EB Activation by the Natural Phenol Pterostilbene
Source: Oxid Med Cell Longev. 2021 Dec 28;2021:7658501. doi: 10.1155/2021/7658501 (PMC8727145; doi:10.1155/2021/7658501)
Supplement: Supplementary Materials — Supplementary Table 1: list of forward and reverse primers used for RT-qPCR analysis. Supplementary Figure 1: signaling pathways involved in TFEB activation. Supplementary Figure 2: Pt triggers autophagy in cultured HeLa cells. Supplementary Figure 3: representative images of WT HeLa cells loaded with MitoSOX™ dye and treated as indicated. Supplementary Figure 4: modulation of cAMP levels is not a key element in Pt-induced AMPK phosphorylation and TFEB migration. Supplementary Figure 5: role of lysosomal Ca2+ in TFEB migration. [file 7658501.f1.pdf]

Multiple mechanisms converging on Transcription Factor EB activation by the natural phenol pterostilbene

Martina La Spina[a,*], Michele Azzolini[a,b, §], Andrea Salmaso[a,b,c], Sofia Parrasia[a], Eva Galletta[a,c] , Marco Schiavone[a,#], Martina Chrisam[d], Andrea Mattarei[e], Giulietta Di Benedetto[b], Andrea Ballabio[f,g,h,i], Natascia Tiso[c], Mario Zoratti[a,b], Lucia Biasutto[a,b,*]

[a] Department of Biomedical Sciences, University of Padova, Padova, Italy;

[b] CNR Neuroscience Institute, Padova, Italy;

[c] Department of Biology, University of Padova, Padova, Italy

[d] Department of Molecular Medicine, University of Padova, Padova, Italy;

[e] Department of Pharmaceutical and Pharmacological Sciences, University of Padova, Padova, Italy;

[f] Telethon Institute of Genetics and Medicine (TIGEM), Pozzuoli, Italy;

[g] Department of Translational Medical Sciences, Section of Pediatrics, Federico II University, Naples, Italy;

[h] Department of Molecular and Human Genetics, Baylor College of Medicine, Houston, TX, USA;

[i] Jan and Dan Duncan Neurological Research Institute, Texas Children Hospital, Houston, TX, USA.

[§] current address: Dept. Physiology and Pharmacology, Karolinska Institutet, Stockholm, Sweden.

[#] current address: Dept. of Molecular and Translational Medicine, University of Brescia, Brescia, Italy.

*: corresponding authors: martina.spina@bio.unipd.it (M.L.S.), lucia.biasutto@cnr.it (L.B.).

Supplementary Material

Supplementary Table 1. *List of forward and reverse primers used for RT-qPCR analysis.*

ACTB: β-Actin; *ATP6V1*: ATPase H+ Transporting lysosomal V1 subunit H; *CTSF:* Cathepsin F; *Gapdh*: Glyceraldehyde-3-Phosphate Dehydrogenase; *MCOLN1*: mucolipin 1; *PPARGC1a*: Peroxisome Proliferator-Activated receptor γ Coactivator 1α; *TFEB*: Transcription Factor EB; *TPP1*: Tripeptidyl Peptidase 1.

All sequences are in the 5’ to 3’ direction.

Gene	Specie	Forward	Reverse
ACTB	human	CTCTTCCAGCCTTCCTTCCT	AGCACTGTGTTGGCGTACAG
ATP6V1	human	GGAAGTGTCAGATGATCCCCA	CCGTTTGCCTCGTGGATAAT
CTSF	human	ACAGAGGAGGAGTTCCGCACTA	GCTTGCTTCATCTTGTTGCC
Gapdh	mouse	TGTGTCCGTCGTGGATCTGA	TTGCTGTTGAAGTCGCAGGAG
MCOLN1	human	TTGCTCTCTGCCAGCGGTACTA	GCAGTCAGTAACCACCATCGG
PPARGC1a	human	AAACAGCAGCAGACAAATGC	TTGGTTTGGCTTGTAAGTGTTGTG
TFEB	human	CCAGAAGCGAGAGCTCACAGAT	TGTGATTGTCTTTCTTCTGCC
Tfeb	mouse	GACTCAGAAGCGAGAGCTAACA	TGTGATTGTCTTTCTTCTGCCG
TPP1	human	GATCCCAGCTCTCCTCAATACG	GCCATTTTTGCACCGTGT

Supplementary Figure 1. *Signaling pathways involved in TFEB activation. For overviews see, e.g., (Settembre, Fraldi et al. 2013, Yoshii and Mizushima 2015, Bento, Renna et al. 2016, Napolitano and Ballabio 2016, Rodney, Pal et al. 2016, Carroll and Dunlop 2017, Rodger, McWilliams et al. 2017, Wang and Zhang 2019). Please note that this is not intended as a complete depiction of all relevant signaling. Furthermore, as this work contributes to show, different branches of the signaling network may have different relevance in different cells. Elements written in bold are those directly investigated in this work.*

This figure was created using images from Servier Medical Art (http://smart.servier.com). Servier Medical Art by Servier is licensed under a Creative Commons Attribution 3.0 Unported License.

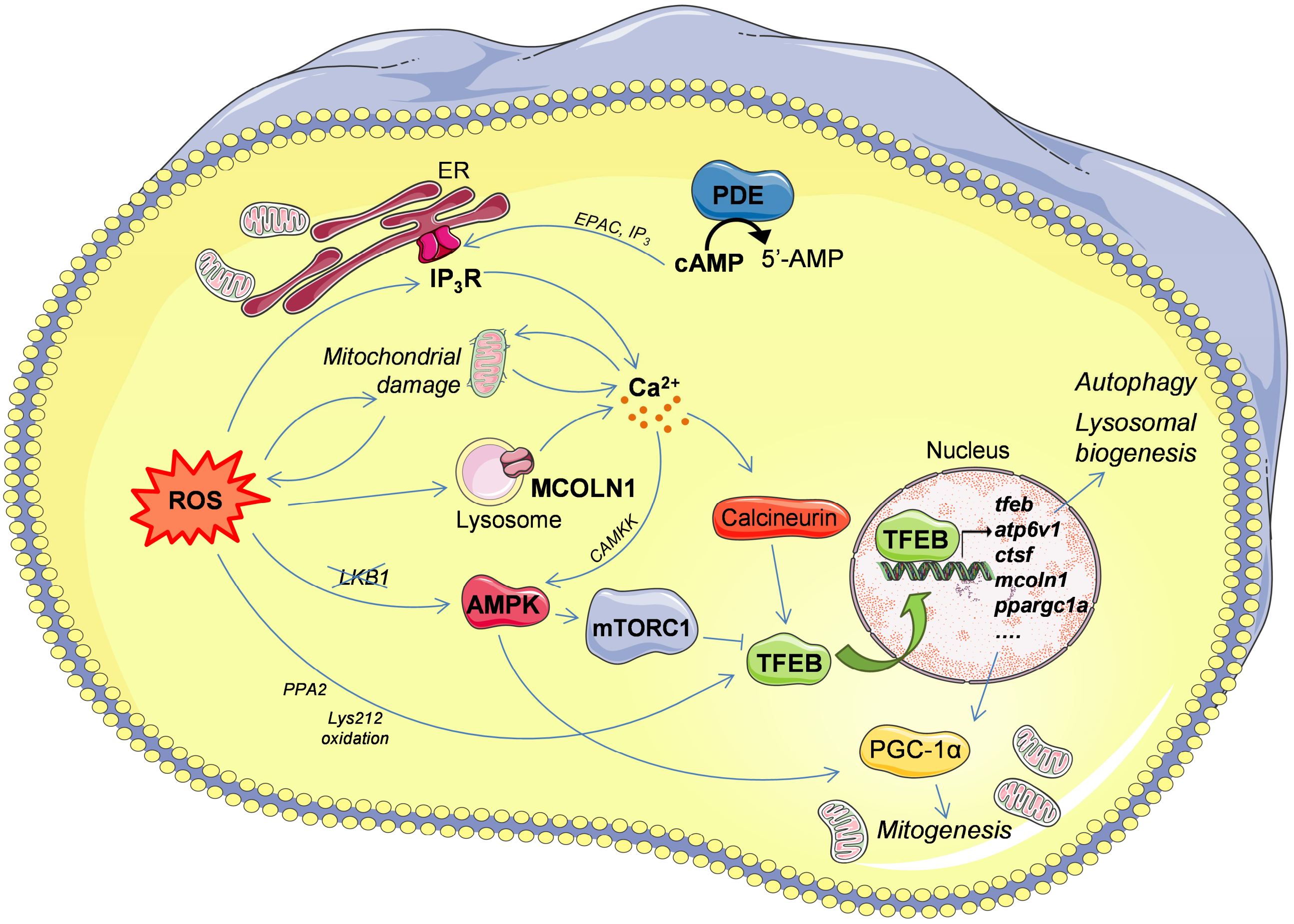

Supplementary Figure 2. *Pt triggers autophagy in cultured HeLa cells.* ***A****: representative Western blot and quantification of the lipidated LC3 in cells treated as indicated. Statistical analysis, performed using Kruskal-Wallis' non-parametric test applying Dunn's correction, shows a significant increase vs control (0.1% DMSO) with 10 µM ($p < 0.001$) and 25 µM ($p < 0.05$) Pt. Shown are relative mean values + SEM; N ≥ 3.* ***B****: representative images and* ***C****: quantification of autophagosomes in HeLa cells treated as indicated. Staining with anti-LC3 antibody; see below for details.*

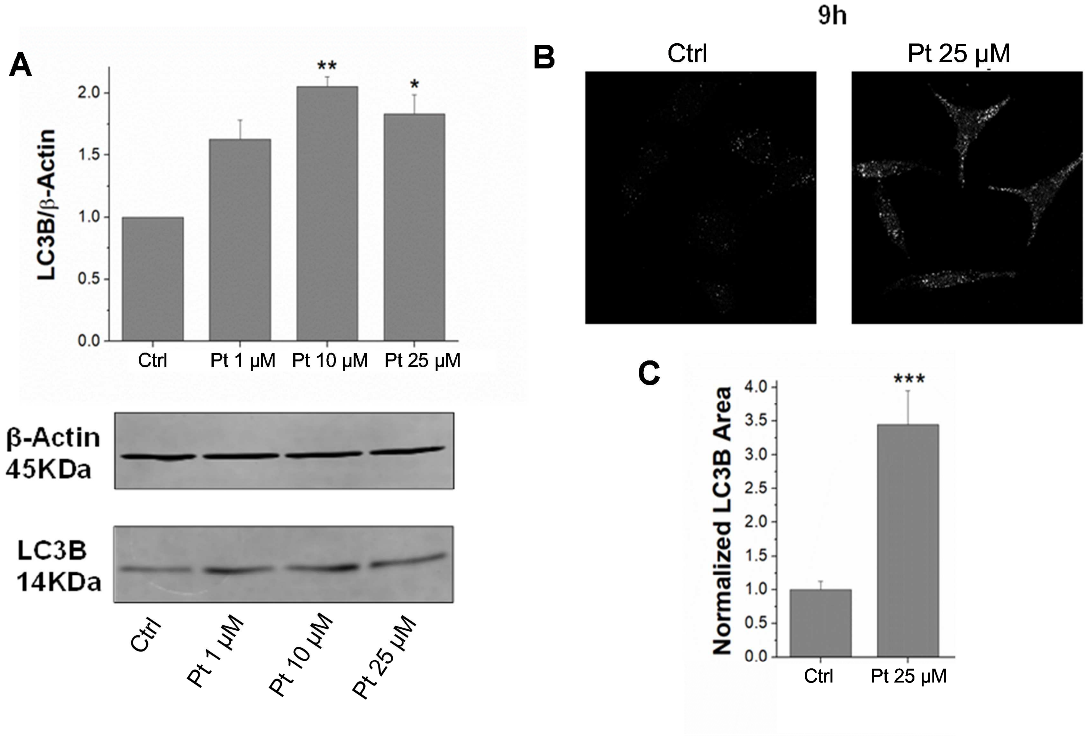

Immunofluorescence and measurement of LC3-positive puncta. WT HeLa cells grown on glass coverslips were incubated with Pterostilbene for 9 hours. After treatment, cells were washed once with 1X PBS and fixed in 1:1 methanol/acetone at −20° C for at least 20 minutes. After fixation, cells were washed once with PBS, incubated with blocking solution (5% goat serum in PBS) for 1 hour and then with α-LC3 primary antibody (1:200, Cell Signaling, Rb, Catalog #3868) in a moist chamber at 4° C overnight. Cells were washed three times with PBS and incubated with a goat α-rabbit secondary antibody, Alexa Fluor488 (1:1000, Thermo Fisher Scientific) at room temperature, in the dark, for 1 hour. Finally, cells were washed three times in PBS and once in dH_2O. Coverslips were mounted onto glass slides with Mowiol mounting medium (Sigma-Aldrich). Cells were imaged with a Leica SP5 confocal system. The number of autophagosomes puncta was calculated using a 3D-object-based method imposing automatically generated fluorescence thresholds on Fiji software. The averaged values were normalized to control (0.1% DMSO treated cells).

Supplementary Figure 3. *Representative images of WT HeLa cells loaded with MitoSOX™ dye and treated as indicated.*

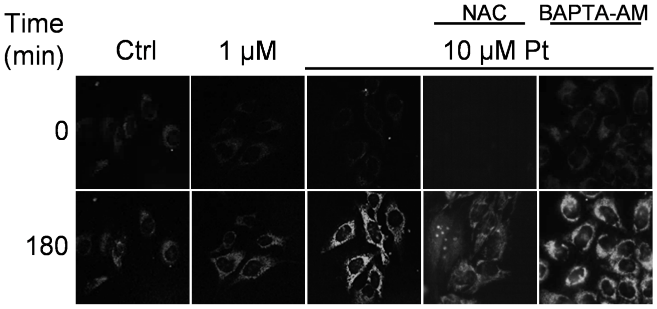

Supplementary Figure 4. *Modulation of cAMP levels is not a key element in Pt-induced AMPK phosphorylation and TFEB migration.* ***A****: time course of cAMP hydrolysis in lysates of HeLa cells treated as indicated (additions at time = 0), as determined by HPLC/UV analysis. The histogram reports the difference in cAMP content of the treated samples compared to control, as measured at 30 min. The difference in the presence of IMBX 100 µM was taken as 100%. N = 6. Error bars: SEM.* ***B****: cAMP levels do not increase significantly in HeLa cells treated with pterostilbene or with IBMX, while they increase upon treatment with forskolin or forskolin + IBMX (ELISA assay).* ***C****: plot of the TFEB-GFP nuclear/cytosolic fluorescence ratio in cell exposed to 25 µM Pt (upper curve; same as in Fig. 1B) or to 25 µM forskolin + 100 µM IBMX. Mean values + SEM. Despite the increase in cAMP levels, forskolin + IBMX did not induce translocation of TFEB-GFP to the nucleus.* ***D*** *and* ***E****: Western Blot analysis of phospho-CREB (S133).* ***D****: 25 µM Pt progressively increases phosphorylation levels of CREB in HeLa cells, with maximal activation after 60-75 minutes.* ***E****: phospho-CREB levels upon 1h-treatment with 1 µM Pt and 10 µM Pt. Mean values + SEM; N ≥ 4. For experimental details see below.*

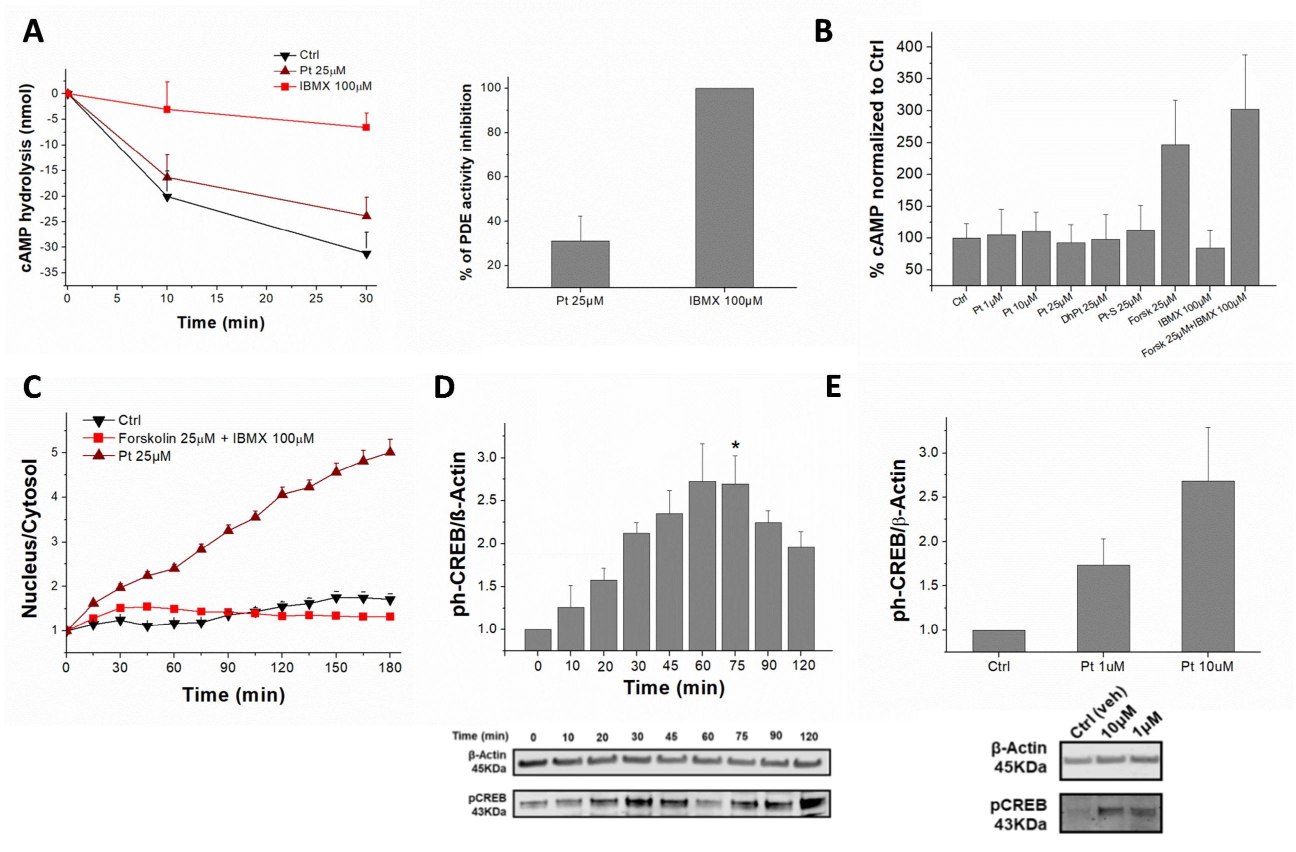

Phosphodiesterases (PDEs) activity assays (Suppl. Fig. 4A). Adherent cells were detached using trypsin (0.05%) and centrifuged (200 *g*, 10 minutes, room temperature). Next, they were rinsed with HBSS, re-suspended in an appropriate buffer (50 mM Tris-HCl, 10 mM $MgCl_2$, pH 8), supplemented with protease inhibitor, at the final density of 3,000,000 cells/mL and sonicated (three times, 10 seconds/each). cAMP to a final concentration of 100 µM was added to the lysate. 150 µL samples of the suspension were collected at 0,

10, 30 and 60 minutes after cAMP addition, boiled for 2 minutes and centrifuged at 12000*g* for 5 minutes. Finally, the supernatant was collected and analyzed with a HPLC/UV (1290 Infinity LC System, Agilent Technologies) by using a reverse phase column (Zorbax RRHD Eclipse Plus C18, 1.8 µm, 50 x 2.1 mm i.d.; Agilent Technologies) and a UV diode array detector (190-500 nm). Solvents A and B were water containing 0.1% trifluoroacetic acid (TFA) and acetonitrile (ACN), respectively. The gradient for B was as follows: 1% for 1 minute, and then from 1% to 100% in 2 minutes. The flow rate was 0.5 mL/min. The eluate was preferentially monitored at 254 nm.

cAMP measurements (Suppl. Fig. 4B). cAMP levels were measured by using a competitive ELISA assay (Cayman Chemicals, prod. #581001). Briefly, WT HeLa cells were seeded on 6-well plates at a density of 300,000 cells/well, and left to adhere and grow in DMEM + 10% FBS for 8 hours. Complete medium was then replaced with DMEM without FBS and Phenol Red (2 mL/well). After 16 hours, 1 mL of the medium was discarded and replaced with 1 mL of DMEM containing one of the treatments at a 2x concentration. Cells were treated for 30 min at 37°C; at the end of the treatment, the medium was discarded, cells were washed with PBS and lysates were prepared and assayed as indicated by manufacturer's instructions.

Cyclic nucleotide levels were also measured by using the specific EPAC1-based FRET sensor H30 described by (Ponsioen, Zhao et al. 2004). Briefly, WT HeLa cells seeded onto a glass coverslip, at 50-70% of confluence, were transiently transfected with the specific probes using the FuGENE-6 transfection reagent (Promega) according to manufacturer's instructions. Imaging experiments were performed 24-48h after transfection. Specifically, cells were maintained in Hepes-buffered Ringer-modified saline (125 mM NaCl, 5 mM KCl, 1 mM Na_3PO_4, 1 mM $MgSO_4$, 5.5 mM glucose, 1 mM $CaCl_2$ and 20 mM Hepes, pH 7.5) at room temperature. Images were captured with an inverted microscope (IX50, 60x NA 1.4 oil immersion objective, Olympus), using custom-made software. FRET fluctuations were measured as changes in the background-subtracted 480/545 nm fluorescence emission intensities on excitation at 430 nm and expressed as either R/R_0, where R is the ratio at time t and R_0 is the ratio at time = 0 s, or $\Delta R/R_0$ where $\Delta R = R-R_0$.

Supplementary Figure 5. *Role of lysosomal* Ca^{2+} *in TFEB migration. Plot of the nucleus/cytosol green fluorescence ratios after the indicated additions or medium exchange at time zero. Mean values + SEM. N ≥ 29 for each time point and condition, observed in at least 3 separate experiments.*

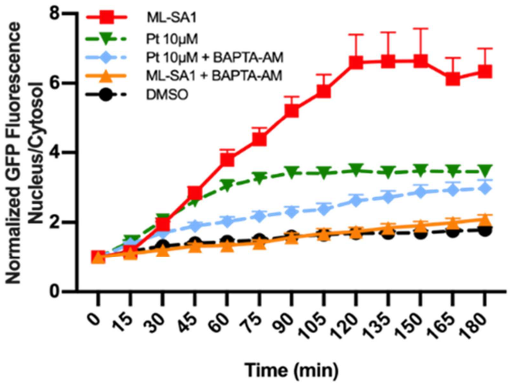

References

Bento, C. F., M. Renna, G. Ghislat, C. Puri, A. Ashkenazi, M. Vicinanza, F. M. Menzies and D. C. Rubinsztein (2016). "Mammalian Autophagy: How Does It Work?" Annu Rev Biochem **85**: 685-713.

Carroll, B. and E. A. Dunlop (2017). "The lysosome: a crucial hub for AMPK and mTORC1 signalling." Biochem J **474**(9): 1453-1466.

Napolitano, G. and A. Ballabio (2016). "TFEB at a glance." J Cell Sci **129**(13): 2475-2481.

Ponsioen, B., J. Zhao, J. Riedl, F. Zwartkruis, G. van der Krogt, M. Zaccolo, W. H. Moolenaar, J. L. Bos and K. Jalink (2004). "Detecting cAMP-induced Epac activation by fluorescence resonance energy transfer: Epac as a novel cAMP indicator." EMBO Rep **5**(12): 1176-1180.

Rodger, C. E., T. G. McWilliams and I. G. Ganley (2017). "Mammalian mitophagy - from in vitro molecules to in vivo models." Febs j.

Rodney, G. G., R. Pal and R. Abo-Zahrah (2016). "Redox regulation of autophagy in skeletal muscle." Free Radic Biol Med **98**: 103-112.

Settembre, C., A. Fraldi, D. L. Medina and A. Ballabio (2013). "Signals from the lysosome: a control centre for cellular clearance and energy metabolism." Nat Rev Mol Cell Biol **14**(5): 283-296.

Wang, Y. and H. Zhang (2019). "Regulation of Autophagy by mTOR Signaling Pathway." Adv Exp Med Biol **1206**: 67-83.

Yoshii, S. R. and N. Mizushima (2015). "Autophagy machinery in the context of mammalian mitophagy." Biochim Biophys Acta **1853**(10 Pt B): 2797-2801.
